# Biological roles and clinical translation of low-density neutrophils in rheumatic, autoimmune and inflammatory diseases: paradigm reconstruction and unresolved questions

**DOI:** 10.3389/fimmu.2026.1932983

**Published:** 2026-09-18

**Authors:** Zhidan Fan, Wenjing Zhu, Panpan Wang, Haiguo Yu

**Affiliations:** Department of Rheumatology, Children’s Hospital of Nanjing Medical University, Nanjing, China

**Keywords:** autoimmune diseases, autoinflammatory diseases, immunological disorders, low-density neutrophils, rheumatic diseases, therapeutic targets

## Abstract

Neutrophils are increasingly recognized as a heterogeneous lineage rather than a uniform population of short-lived innate effector cells. Low-density neutrophils (LDNs; often termed low-density granulocytes, LDGs) are operationally defined by their co-sedimentation with peripheral blood mononuclear cells after density-gradient centrifugation, but this physical definition encompasses cells with different maturation states, origins and functions. In rheumatic and autoimmune diseases, expansion of the low-density compartment is associated with disease activity, autoantibody titers, interferon signatures and organ involvement. Mechanistic studies implicate enhanced neutrophil extracellular trap (NET) formation, oxidized mitochondrial DNA, platelet-neutrophil interactions, inflammasome activation and endothelial injury in disease amplification. However, LDNs can also be detected in healthy individuals, and immunoregulatory low-density neutrophil states occur in other biological contexts, underscoring that low density is not synonymous with pathogenicity. This Review critically integrates current evidence on LDN origin, heterogeneity, disease associations and clinical translation. Particular emphasis is placed on the unresolved distinction between correlation and causation, the limitations of density-based nomenclature, and the evidentiary gap between mechanistic observations and therapeutic claims. We distinguish findings demonstrated in rheumatic disease from strategies extrapolated from general neutrophil biology or tumor-associated PMN-MDSCs, and outline the methodological and experimental steps required for molecularly defined biomarkers and subset-specific interventions.

## 1 Introduction

Advances in single-cell transcriptomics, high-dimensional cytometry and functional proteomics have established that neutrophils comprise multiple developmental and functional states rather than a homogeneous terminally differentiated population (1, 2). Low-density neutrophils (LDNs), also referred to as low-density granulocytes (LDGs), are operationally identified by their recovery in the peripheral blood mononuclear cell (PBMC) fraction after density-gradient centrifugation and have become a prominent example of this heterogeneity.

LDNs were first described in 1986 in the PBMC fraction of patients with systemic lupus erythematosus (SLE), rheumatoid arthritis (RA) and acute rheumatic fever (3). Subsequent work has detected low-density neutrophil populations in autoimmune disease, infection, cancer, pregnancy and healthy individuals. This broad distribution highlights an important limitation of current terminology: LDN/LDG denotes a physical separation phenotype, whereas terms such as immature neutrophil and polymorphonuclear myeloid-derived suppressor cell (PMN-MDSC) imply developmental or functional properties and should not be used interchangeably without supporting evidence (4, 5).

Current evidence supports at least two major, non-exclusive routes into the low-density compartment: premature release of immature neutrophils during altered granulopoiesis and density reduction of mature neutrophils following activation, degranulation or metabolic reprogramming (6–8). Their relative contributions vary with disease context and inflammatory state, making LDNs a heterogeneous population rather than a single lineage (9, 10).

In rheumatic diseases, circulating LDN abundance and LDN-associated molecular signatures are repeatedly associated with disease activity, autoantibodies, type I interferon signaling and organ damage (11–16). Mechanistic studies further implicate NET formation, platelet-neutrophil interactions, inflammasome activation and endothelial injury. Nevertheless, most human evidence remains observational; therefore, the extent to which specific LDN states initiate disease, amplify established inflammation or simply reflect inflammatory burden remains unresolved (17).

A recent review in Frontiers in Immunology by Yin et al. summarized the biological characteristics of LDNs, their roles across rheumatic diseases and their potential therapeutic value (16). The present Review is intended to complement, rather than duplicate, that disease-oriented overview by focusing on the conceptual and evidentiary problems that currently limit interpretation and translation of the LDN literature.

Specifically, we examine how the field can move from a density-defined descriptive phenotype toward molecularly resolved, causally validated and clinically actionable neutrophil states. We therefore emphasize five linked issues: nomenclature and definition, cellular origin, functional heterogeneity, correlation versus causation, and the evidence hierarchy underlying biomarker and therapeutic claims. Disease-specific findings are discussed in this framework, and therapeutic strategies are explicitly separated into observations demonstrated in rheumatic disease, preclinical evidence, and hypotheses extrapolated from general neutrophil or tumor/MDSC biology.

Accordingly, the added value of this Review is its critical framework rather than a broader disease catalogue: we integrate emerging single-cell, multi-omic, spatial, cell-interaction and immunometabolic concepts with an explicit assessment of where the evidence remains descriptive, correlational or extrapolated. This positioning is intended to complement recent disease-oriented reviews and to define experimentally testable priorities for the next phase of LDN research.

## 2 Biological definition, origin and functional heterogeneity of low-density neutrophils

Neutrophils have long been defined as terminally differentiated, functionally homogeneous innate immune effector cells, with phagocytic killing, degranulation and neutrophil extracellular trap (NET) formation as core effector modalities, serving as the first line of defense in acute infection. Over the past three decades, however, advances in separation technologies and the rise of multi-omics approaches have progressively unveiled the profound heterogeneity and functional plasticity of neutrophils, completely overturning the classical dogma of a “single lineage, single function” (10, 18, 19). Among the identified subsets, low-density neutrophils (LDNs) — a population of neutrophils that co-sediment with the peripheral blood mononuclear cell (PBMC) layer after Ficoll density gradient centrifugation — have become one of the most controversial and translationally valuable research directions in neutrophil biology, because they specifically expand under various pathological conditions, including autoimmunity, chronic infection and cancer, and possess the dual functional potential for both pro-inflammatory injury and immune regulation (1, 2, 14).

In rheumatic diseases, the abnormal expansion of LDNs is closely associated with disease activity, the extent of organ damage and long-term prognosis. Through multiple mechanisms, including NET release, cytokine secretion and endothelial injury, they participate in the initiation and amplification of autoimmune inflammation and are regarded as key nodes linking aberrant innate immunity with disordered adaptive immunity (16, 20, 21). Nevertheless, the field still faces fundamental challenges: blurred definitional boundaries, controversies regarding their origin, contradictory functional phenotypes, and a lack of reproducibility across studies stemming from non-standardized methodologies — all of which severely impede progress toward clinical diagnosis and targeted therapy (4, 22). This chapter systematically dissects the research progress on LDNs across four dimensions — definitional tracing, the origin debate, phenotypic heterogeneity and functional polymorphism — and critically reflects on the methodological limitations and conceptual pitfalls in current research, thereby laying a theoretical framework for deciphering their pathological roles in rheumatic diseases.

### 2.1 Definition, nomenclature and limitations of density-based classification

The definition of LDNs has been strongly methodology-driven from the beginning: it is a descriptive concept based on density-gradient separation rather than a biological classification grounded in lineage or function. In 1986, granulocyte-like cells in the PBMC fraction were termed low-density granulocytes (LDGs); subsequent work established that neutrophils comprise the dominant population, and “low-density neutrophil (LDN)” became the preferred operational term. In historical and disease-specific literature, LDN and LDG are often used for overlapping density-defined populations (11, 20).

With the rise of myeloid-derived suppressor cell (MDSC) research, polymorphonuclear MDSCs (PMN-MDSCs) were also found to exhibit low density and segmented nuclear morphology and to expand under inflammatory and tumor conditions, leading to long-standing confusion among the three concepts — LDNs, LDGs and PMN-MDSCs — which have even been treated as different names for the same cell population in some studies (22, 23). This conceptual conflation is not a trivial issue of terminology; it fundamentally reflects the inherent deficiencies of the current classification system for neutrophil subsets:

- Limitations of the separation method: Ficoll density gradient centrifugation sorts cells based solely on the single physical parameter of buoyant density and cannot distinguish among the different biological mechanisms that lead to decreased density. Immature neutrophils exhibit lower density due to reduced cytoplasmic granule content and incomplete nuclear segmentation; mature neutrophils can decrease in density because of degranulation, cytoplasmic vacuolization or lipid metabolic reprogramming upon inflammatory stimulation; and some activated eosinophils and basophils may also contaminate the low-density fraction (4, 8).
- Heterogeneity of experimental procedures: The Ficoll concentration, centrifugal speed, temperature control and sample resting time vary considerably across laboratories, directly resulting in markedly different cellular compositions of the isolated LDN fractions. This is the core methodological root of the contradictory functional phenotypes reported for LDNs in different studies — some laboratories isolate a fraction dominated by immature precursor cells, whereas others obtain predominantly activated mature neutrophils, two populations with inherently distinct functional characteristics (14, 22).

In response to this chaos, the 2021 international consensus on neutrophil nomenclature explicitly stated that “low-density neutrophil” should be used only as a descriptive term referring to the total neutrophils located in the PBMC layer after density gradient centrifugation, without presuming a specific functional phenotype; in the absence of specific molecular markers, it is impermissible to directly equate LDNs with PMN-MDSCs (22). This consensus establishes a baseline for standardization, but to date, specific surface markers that can accurately distinguish different LDN subsets are still lacking, and no globally unified isolation and identification standard has been formulated. This research landscape of “definition preceding mechanism” is the primary bottleneck that LDN research must overcome. The purification procedure and core immunophenotypic characteristics of LDNs are systematically illustrated in **Figure 1**.

**Figure 1:**
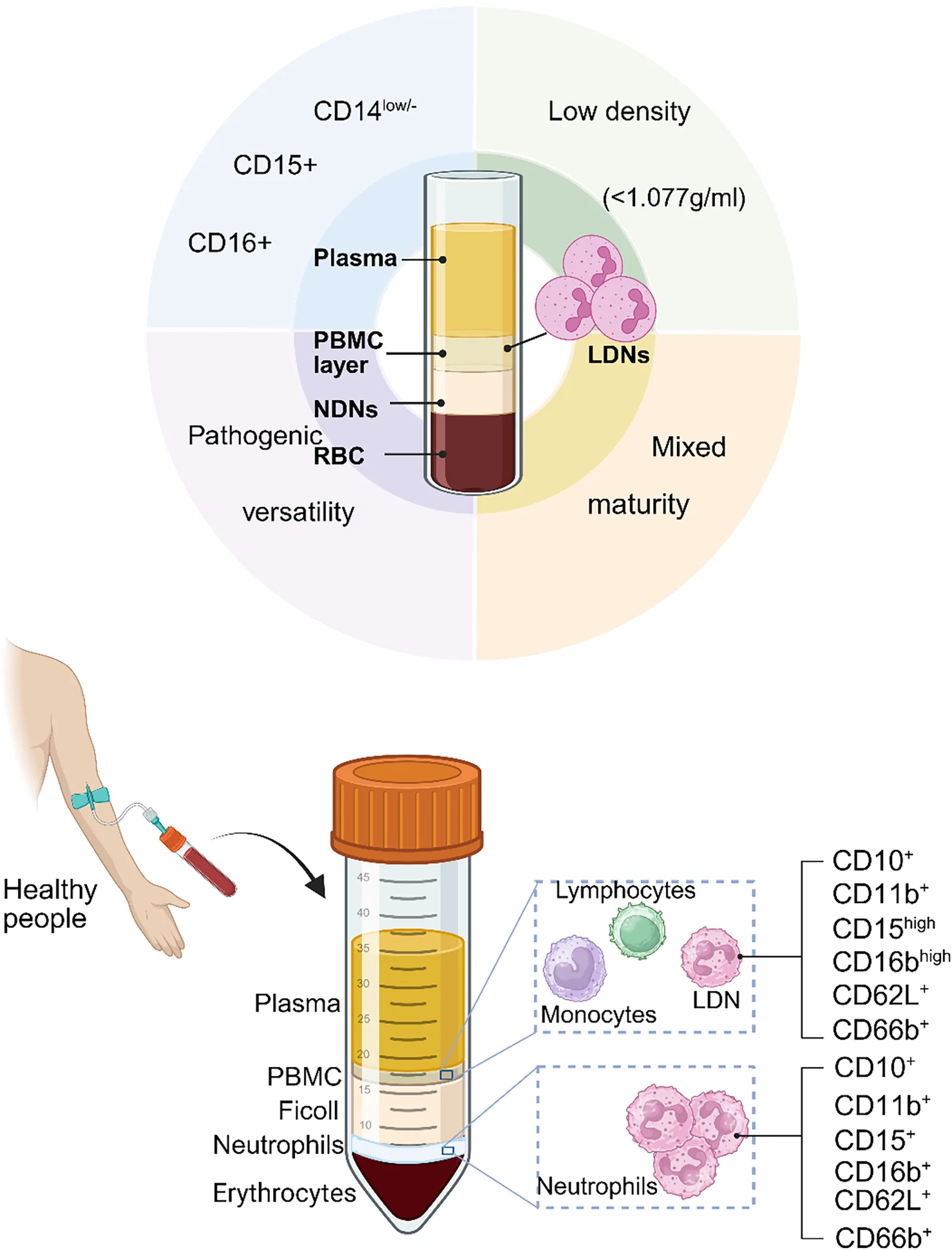
Purification workflow and immunophenotypic characteristics of low-density neutrophils. Whole peripheral blood from healthy individuals is separated via Ficoll density gradient centrifugation, with low-density neutrophils (LDNs) sedimenting within the PBMC layer owing to their density <1.077 g/mL. The circular inset highlights core biological traits of LDNs: low buoyant density, heterogeneous maturation stages, and diverse functional states in health and autoimmunity. Surface immunomarker panels compare phenotypic profiles of LDNs and conventional neutrophils; LDNs display CD14low alongside upregulated CD15high and CD16high, sharing CD10, CD11b, CD62L and CD66b expression with normal-density neutrophils. These phenotypic features illustrate the limitations of assigning function from density or a small marker panel alone. **Figure 1** Schematic diagram created with BioRender.com.

Terminology used in this Review: “LDN” is used as the preferred operational term for neutrophils recovered in the low-density/PBMC fraction. “LDG” is retained only when referring to the terminology of an original study. “Normal-density neutrophil (NDN)” is used for the conventional granulocyte-density fraction. “PMN-MDSC” is reserved for low-density neutrophil-like populations with demonstrated immunosuppressive activity and is not treated as a synonym for LDN.

### 2.2 Cellular origins: emergency granulopoiesis and peripheral reprogramming

The cellular origin of LDNs is a core scientific question that continues to be debated, with two mainstream hypotheses: the “bone marrow origin” hypothesis and the “peripheral reprogramming” hypothesis. Their fundamental divergence lies in whether LDNs are precursor cells released prematurely during neutrophil development or a functional state adopted by mature neutrophils that undergo reprogramming in the peripheral inflammatory milieu. The answer to this question directly determines our understanding of the pathological role of LDNs and the design of intervention strategies. Altered bone marrow granulopoiesis is one plausible contributor to peripheral LDN accumulation in rheumatic autoimmune diseases (**Figure 2**), alongside peripheral reprogramming of mature neutrophils.

**Figure 2:**
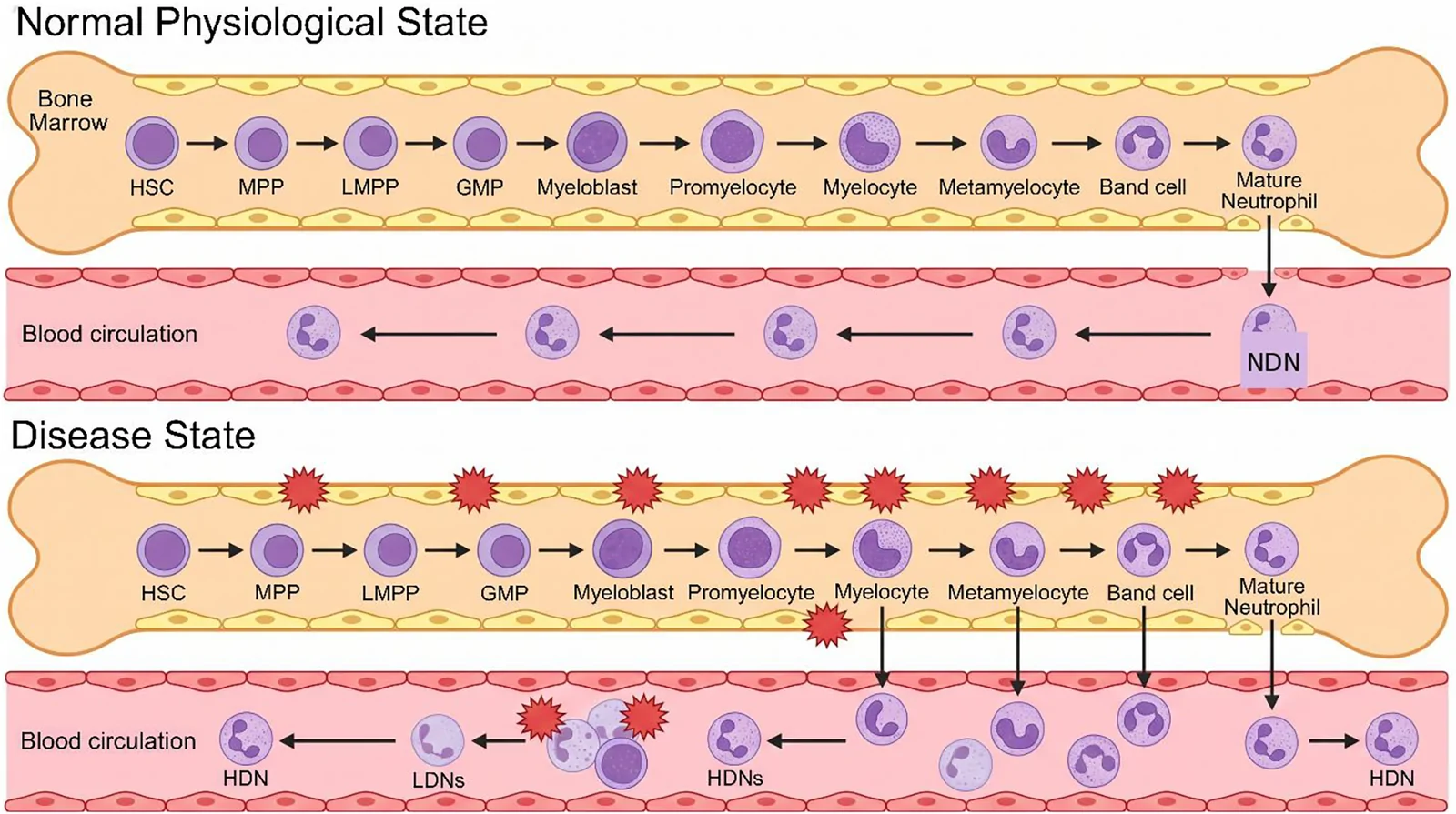
Altered bone marrow granulopoiesis mediates peripheral LDN accumulation under autoimmune pathological conditions. Neutrophils undergo stepwise bone-marrow maturation from HSCs via MPP, LMPP, GMP and downstream precursor intermediates; only fully differentiated neutrophils enter circulation as NDNs under physiological steady state. In rheumatic diseases, inflammatory insults perturb multiple stages of marrow granulocytic differentiation. Immature neutrophil progenitors prematurely exit bone marrow into peripheral blood, generating the heterogeneous circulating LDN population that coexists with mature NDNs. This hematopoiesis disturbance largely accounts for the expanded LDN fractions observed in patients with autoimmune rheumatic illnesses. **Figure 2** Schematic diagram created with BioRender.com.

#### 2.2.1 Bone marrow origin and emergency granulopoiesis

Early studies on SLE found that patient-derived LDNs exhibit immature morphological features such as reduced nuclear segmentation and sparse cytoplasmic granules, leading to the proposal that LDNs result from aberrant bone marrow granulopoiesis with premature release of immature neutrophils into the peripheral circulation (20, 24). Subsequent transcriptomic studies further confirmed that LDNs from SLE patients highly express genes associated with early neutrophil development (e.g., MPO, ELANE, PRTN3) and cell cycle regulation, and their transcriptional profiles substantially overlap with those of bone marrow metamyelocytes and band neutrophils (6, 25).

This hypothesis has received broad pathophysiological support: during acute stress states such as sepsis and severe bacterial infection, the organism initiates an “emergency granulopoiesis” program, accelerating bone marrow granulocyte proliferation and differentiation, with large numbers of immature neutrophils prematurely released into the peripheral circulation, directly causing a significant increase in LDN proportions (5, 26); in chronic autoimmune diseases such as SLE, sustained stimulation of bone marrow hematopoietic stem/progenitor cells by type I interferon, GM-CSF and other cytokines leads to aberrant activation of granulopoietic pathways and increased release of immature neutrophils, which become an important source of LDNs (2, 27).

#### 2.2.2 Peripheral reprogramming: activation and density transition of mature neutrophils

The “peripheral reprogramming” hypothesis poses a fundamental challenge to the bone marrow origin model. This hypothesis posits that LDNs are not a developmentally distinct cell lineage, but rather a functional state arising when mature neutrophils, upon stimulation by cytokines, immune complexes and damage-associated molecular patterns (DAMPs) in the peripheral inflammatory microenvironment, undergo activation, degranulation and metabolic reprogramming, ultimately leading to reduced cell density (1, 8).

Core evidence supporting this hypothesis includes:

- Existence of LDNs in healthy individuals: A landmark 2020 study by Hassani et al. rigorously isolated the 20% lowest-density and 20% highest-density neutrophils from healthy human peripheral blood. They found that healthy LDNs are predominantly mature segmented neutrophils whose phagocytic capacity, ROS production and other functions do not differ significantly from those of normal-density neutrophils (NDNs), with only slightly impaired chemotaxis and no obvious immature transcriptional signature (8). This result directly demonstrates that mature neutrophils can exhibit a low-density phenotype even in the absence of pathology; LDNs are not exclusive to pathological states.
- Feasibility of *in vitro* induction: Co-culturing healthy mature neutrophils with GM-CSF, IFN-α, TNF-α or immune complexes can successfully induce a decrease in their density, yielding an activated phenotype and functional characteristics similar to those of *in vivo* LDNs (2, 6).
- Causal link between activation and density: LDNs from patients with rheumatic diseases universally upregulate activation markers such as CD11b and CD66b and degranulate to a significantly greater extent than NDNs; the release of granular contents directly reduces cell specific gravity, providing a direct physical mechanism for density reduction (20, 21).

#### 2.2.3 A mixed-origin model of the low-density compartment

Critically, the two hypotheses are not mutually exclusive but coexist in varying proportions depending on the disease context and stage. LDNs are fundamentally not a single homogeneous cell subset but a mixed population comprising both bone marrow-derived immature neutrophils and peripherally activated mature neutrophils, with their relative proportions determined by the intensity, duration and type of inflammation:

- In strong acute inflammation such as infection and sepsis, emergency granulopoiesis dominates, and LDNs are primarily immature cells;
- In chronic autoimmune diseases such as SLE and RA, both aberrant bone marrow development and peripheral activation-induced reprogramming operate simultaneously, yielding a mixed population of immature and activated mature cells;
- In healthy physiological states, LDNs consist mainly of mildly activated mature neutrophils in the circulation, possibly participating in immune surveillance and inflammatory homeostasis maintenance (8, 14).

A widespread pitfall in the current field is that most studies still analyze LDNs as a single population for functional and mechanistic investigations, ignoring their internal origin-based heterogeneity. This research paradigm inevitably leads to one-sided and irreproducible conclusions, and represents a deep-seated reason why LDN research has struggled to achieve breakthroughs for many years.

### 2.3 Shared molecular mechanisms and context-dependent LDN functions

Physiological and pathological remodeling drives the formation of heterogeneous LDN subsets with distinct immunophenotypic signatures (**Figure 3**).

**Figure 3:**
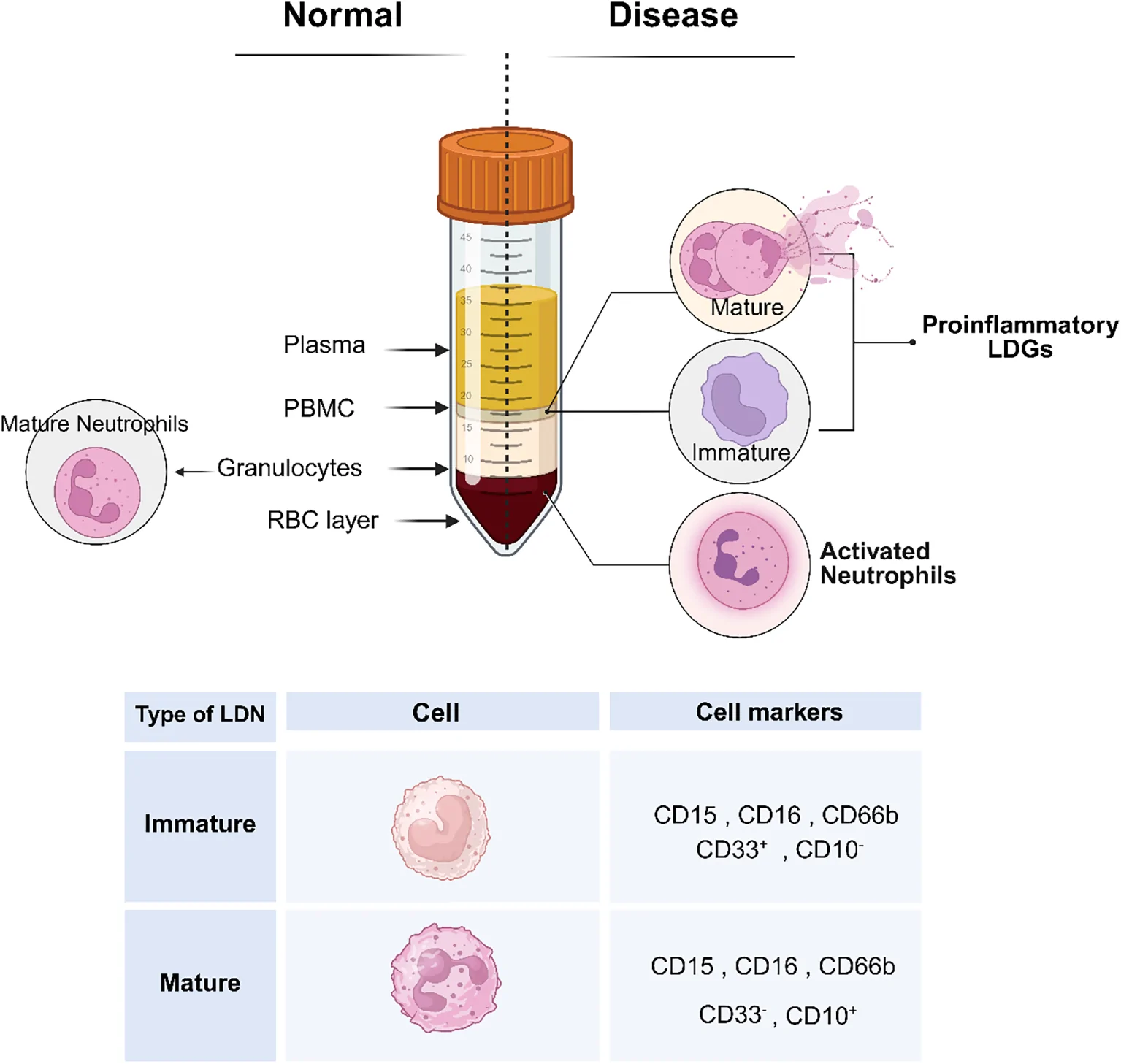
Heterogeneous subsets and immunophenotypic signatures of low-density neutrophils (LDNs) under physiological and autoimmune pathological states. Under normal physiological conditions, conventional mature neutrophils sediment within the granulocyte fraction below the PBMC layer following Ficoll density gradient centrifugation of peripheral blood. In the setting of rheumatic and systemic autoimmune disorders, neutrophil populations undergo phenotypic and maturational remodeling, and diversified low-density granulocytes (LDGs; historically used for overlapping density-defined populations) migrate into the PBMC layer. Disease-associated pro-inflammatory LDN pools may comprise three distinct cellular constituents: mature neutrophils, immature neutrophil precursors, and aberrantly activated mature neutrophils. The appended tabular panel further stratifies LDNs into immature and mature subgroups according to maturation status, and delineates their discriminative surface marker profiles. Immature LDN subsets are characterized by positive CD33 expression alongside CD10^-^, with shared expression of CD15, CD16 and CD66b relative to mature LDNs; mature LDNs conversely exhibit CD33^-^ and CD10^+^ immunophenotypes. Such maturation-associated marker disparities underpin the functional heterogeneity of LDNs, fueling persistent inflammation, tissue injury and diverse immunopathological manifestations across a spectrum of autoimmune rheumatic diseases. **Figure 3** Schematic diagram created with BioRender.com.

Functional studies of LDNs have produced context-dependent and sometimes conflicting results: in autoimmune diseases they are generally defined as pro-inflammatory pathogenic effector cells, whereas in cancer and chronic infection they frequently exhibit pronounced immunosuppressive functions. This functional paradox is essentially the combined result of their origin-based heterogeneity and microenvironment-dependent plasticity.

#### 2.3.1 Shared NET–nucleic acid–IFN-I and tissue-injury pathways

Across rheumatic and autoimmune diseases, the most reproducible shared effector axis is enhanced NET formation coupled to the release of immunostimulatory nuclear and mitochondrial material. LDN-derived NETs can expose modified autoantigens, deliver oxidized mitochondrial DNA and other nucleic acids to plasmacytoid dendritic cells, and amplify type I interferon signaling (2, 20, 24, 28, 29). This NET–nucleic acid–IFN-I circuit should therefore be treated as a cross-disease mechanism rather than re-described independently in every disease section. Its pathogenic weight varies by disease, tissue and LDN state, and most human studies do not establish that this pathway is initiated uniquely by LDNs rather than by other activated neutrophil populations.

A second shared layer is multicellular tissue injury. NET components and neutrophil granule proteins can damage endothelium, activate macrophage inflammasomes, promote thrombosis, and alter T- and B-cell responses (21, 30–32). Platelet–neutrophil interactions and inflammatory cytokines can further reinforce these states. These mechanisms are summarized here once; the disease-specific sections below therefore focus on what is distinctive in each condition—such as SLE-specific oxidized mitochondrial DNA and platelet TLR7 signaling, RA-specific citrullinated autoantigens and synovial interactions, or psoriasis-specific skin homing and IL-36 coupling.

#### 2.3.2 Immunoregulatory states, PMN-MDSCs and microenvironmental dependence

In stark contrast to the pro-inflammatory phenotype, within the tumor microenvironment low-density neutrophils often display classical immunosuppressive functions, inhibiting CD8^+^ T-cell proliferation and cytotoxic function through mechanisms such as the production of reactive oxygen species, arginase 1 (ARG1) and TGF-β — i.e., the core features of PMN-MDSCs (33, 34). This functional paradox implies that LDNs are not a fixed lineage but a highly plastic functional state, whose ultimate phenotype is entirely determined by the prevailing microenvironmental signals.

A critical open question is: do immunoregulatory LDN subsets exist in rheumatic diseases? Existing evidence suggests that a certain proportion of LDNs can still be detected in patients during disease remission, and their pro-inflammatory activity is significantly reduced, suggesting a possible role in inflammation resolution and immune homeostasis restoration (11, 16). However, owing to the lack of specific molecular markers, it is currently impossible to precisely isolate immunoregulatory subsets from the total LDN pool or to verify their role in disease outcome.

The relationship among immature LDNs, mature activated LDNs and PMN-MDSCs should therefore be viewed as overlap among three different classification axes rather than as a linear hierarchy. “Immature” denotes developmental stage, “low density” denotes a biophysical separation phenotype, and “PMN-MDSC” denotes suppressive function. A cell can satisfy more than one of these descriptors, but none can be inferred from another. This distinction is essential when comparing studies that use morphology, density, surface phenotype or T-cell suppression as non-equivalent entry criteria (22, 23). A conceptual comparison of immature, mature/activated, and PMN-MDSC-like low-density neutrophil populations is summarized in **Table 1**.

**Table 1:** Conceptual comparison of immature, mature/activated and PMN-MDSC-like low-density neutrophil populations.

Feature	Immature low-density neutrophils	Mature/activated low-density neutrophils	PMN-MDSC-like low-density neutrophils
Primary descriptor	Developmental stage + low density	Activation/state change + low density	Suppressive function + neutrophil phenotype; often low density
Proposed origin	Emergency/altered granulopoiesis with premature release	Peripheral activation, degranulation, platelet binding or metabolic reprogramming	Context-dependent myeloid differentiation/activation; especially tumor or suppressive inflammatory environments
Typical features	Band/metamyelocyte morphology; lower CD10; developmental granule genes may be enriched	Segmented morphology; activation/degranulation markers; phenotype varies by disease	Requires demonstrable suppression of T-cell or other immune-cell function; density alone is insufficient
Representative evidence	SLE and RA developmental signatures (6, 7, 25, 35)	Healthy low-density mature neutrophils (8); SLE CD98-high/CD177-high states (36–38)	Tumor-associated suppressive neutrophils and multi-omic analyses (23, 33, 34)
Key uncertainty	Whether immature cells are causal, amplifying or simply reflect inflammatory granulopoiesis	Whether low density is stable, reversible or a marker of recent activation	Relationship to rheumatic-disease LDNs and whether suppressive subsets exist *in vivo* in specific rheumatic contexts

From an evolutionary perspective, the functional duality of LDNs is intrinsically reasonable: in acute infection, the rapid replenishment of immature LDNs can enhance antimicrobial defense; during the resolution phase of inflammation, regulatory LDNs can prevent immunopathological damage; whereas in chronic autoimmune diseases, the normal functional balance is disrupted, with pro-inflammatory subsets becoming dominant and potentially sustaining tissue injury. This characteristic of “distinct functional states hidden beneath the same physical phenotype” represents the most fundamental biological essence of LDNs, and is precisely the most easily overlooked point in current research. The context-dependent dichotomous immunomodulatory functions of LDNs in autoimmune inflammation, cancer and pregnancy are summarized in **Figure 4**.

**Figure 4:**
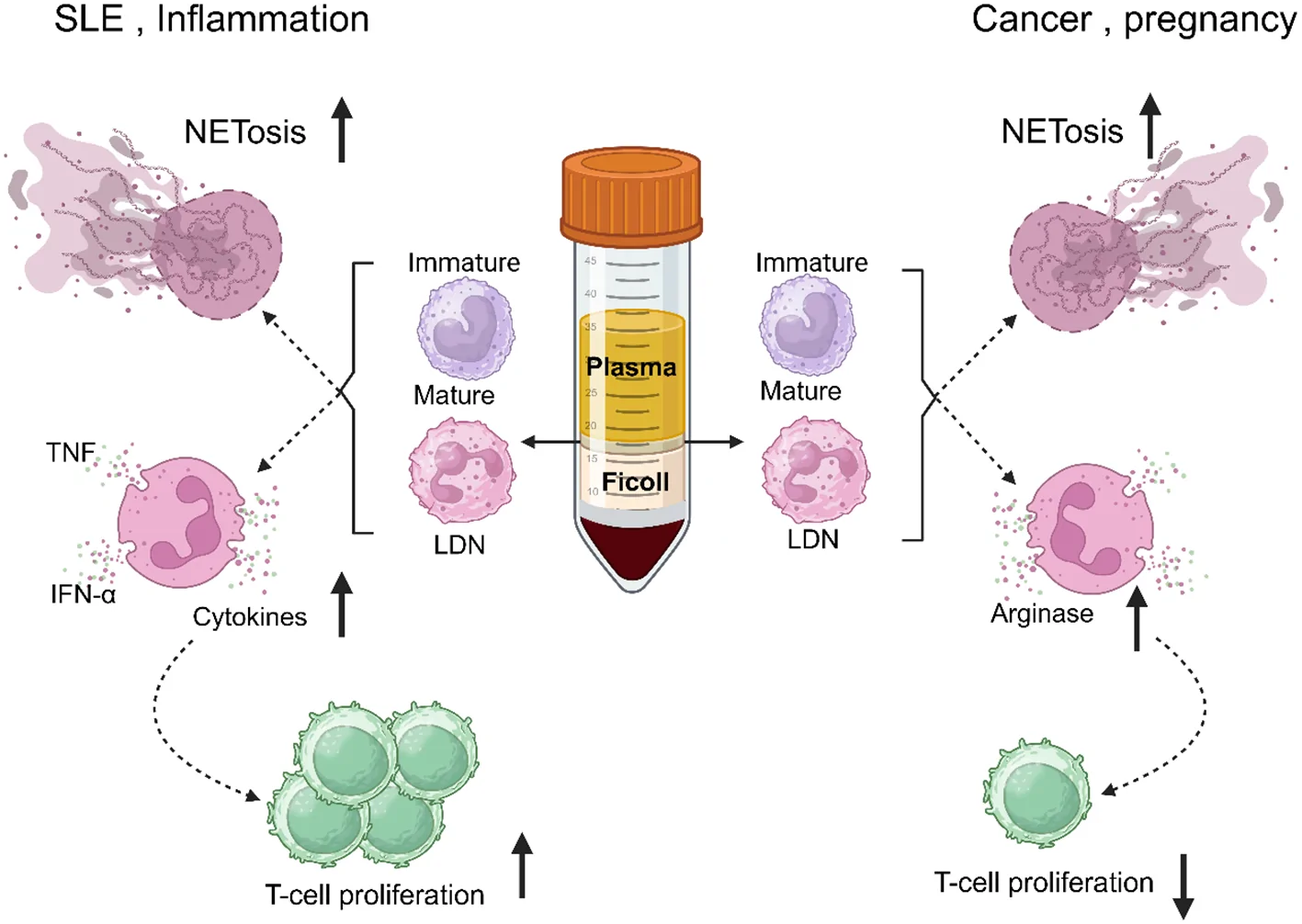
Dichotomous regulatory activities of LDNs in SLE-related inflammation versus cancer and pregnancy settings. Isolated via Ficoll density separation, mature and immature LDN subsets display opposing immunomodulation depending on pathological contexts. In SLE-associated inflammatory conditions, augmented LDN NETosis facilitates TNF and IFN-α secretion to boost T-cell proliferation and exacerbate autoinflammation. By contrast, in cancerous tissues and pregnancy, LDNs upregulate arginase production alongside increased NETosis; released arginase restrains T-cell proliferation to establish immunosuppression. This environment-dependent functional plasticity renders LDNs context-dependent contributors to the distinct immune landscapes of rheumatic autoimmune disorders and other physiological/pathological states. **Figure 4** Schematic diagram created with BioRender.com.

### 2.4 Emerging technologies redefining LDN heterogeneity

Single-cell and multi-omic approaches are beginning to replace bulk low-density measurements with molecularly resolved neutrophil states. Transcriptomic, epigenomic and proteomic studies in SLE have identified substantial intra-LDN diversity, while recent work has described CD98-high metabolically flexible and CD177-high vascular-associated LDN phenotypes (36, 37). Multi-omic analyses of immunosuppressive neutrophils further emphasize that transcriptional similarity or low density alone is insufficient to assign PMN-MDSC identity without functional testing (23). These observations support classification by convergent molecular, developmental and functional features rather than by density alone.

Spatial and cell-interaction technologies add a second dimension by asking where these states act and with which partners. Paired blood-tissue single-cell profiling, spatial transcriptomics and multiplex imaging can determine whether circulating LDN states correspond to neutrophils in kidney, synovium, skin or vascular lesions. Existing mechanistic studies already illustrate the importance of multicellular context: platelet TLR7 signaling promotes platelet-neutrophil complex formation and low-density transition in lupus nephritis (39), whereas IL-1β-dependent fibroblast-neutrophil crosstalk can induce LDN-like states in SLE skin (40). Computational cell-cell communication inference will therefore require validation by spatial colocalization and perturbation experiments rather than interpretation from ligand-receptor signatures alone.

Immunometabolic profiling is also reshaping the field. The CD98-high LDN state links amino-acid transport and metabolic flexibility to inflammatory behavior (36), while mitochondrial ROS and oxidized mitochondrial DNA connect metabolic stress with NET-associated interferogenic activity in SLE (29). However, metabolic state should not be equated with lineage. Time-resolved sampling, trajectory analysis, paired bone-marrow/blood datasets and, where feasible, lineage-tracing or barcoding approaches are needed to determine whether molecular states represent stable developmental branches or reversible activation programs. These technologies are therefore most informative when integrated with functional assays and longitudinal disease sampling.

### 2.5 Methodological and standardization priorities

The field now requires measurable milestones rather than a general call for standardization. A staged roadmap is proposed below.

Milestone 1 — Harmonized pre-analytics and isolation. Multicenter studies should standardize and report anticoagulant, time from phlebotomy to processing, temperature, density medium, centrifugation force/time, braking conditions, cell recovery and purity. These variables should form a minimum reporting checklist.

Milestone 2 — Minimum phenotyping and nomenclature criteria. LDN should remain an operational density term; maturation state, activation state and suppressive function should be reported as separate dimensions. A consensus minimum flow-cytometry panel should include lineage confirmation, maturation markers and activation/degranulation markers, with PMN-MDSC terminology reserved for populations with demonstrated suppressive function.

Milestone 3 — Cross-platform molecular definition. Single-cell transcriptomics, surface proteomics, epigenomics and metabolomics should identify reproducible molecular states, followed by conversion of discovery signatures into practical flow-cytometric or targeted molecular panels that can be tested without relying solely on Ficoll density.

Milestone 4 — Inter-laboratory reproducibility and reference materials. Candidate panels and gating strategies should undergo blinded ring studies across centers, using shared control samples or reference materials, predefined quality-control metrics and reporting of inter-assay coefficients of variation.

Milestone 5 — Biological validation across compartments and time. Paired blood–bone marrow and blood–tissue studies, longitudinal sampling across flare and remission, and spatial profiling of kidney, synovium, skin and vascular lesions should determine whether circulating molecular states correspond to tissue-relevant neutrophils and whether they represent stable lineages or reversible activation states.

Milestone 6 — Clinical qualification. Only after analytical reproducibility and biological validity are established should disease-, age- and treatment-specific reference ranges be derived, prospective biomarker cut-offs tested, and LDN-related endpoints incorporated into interventional trials. This sequence would move the field from a density-defined research phenotype toward a clinically interpretable, molecularly resolved classification. 

## 3 Disease-specific evidence in rheumatic, autoimmune and autoinflammatory diseases

Organization of the disease-specific section. The NET–nucleic acid–IFN-I axis, endothelial injury, inflammasome activation and general adaptive-immune interactions are summarized in Section 2.3 and schematically illustrated in **Figure 5**. The subsections below emphasize disease-specific phenotypes, upstream regulators, tissue contexts, clinical associations and evidence limitations; shared pathways are mentioned only when a disease-specific extension is informative.

**Figure 5:**
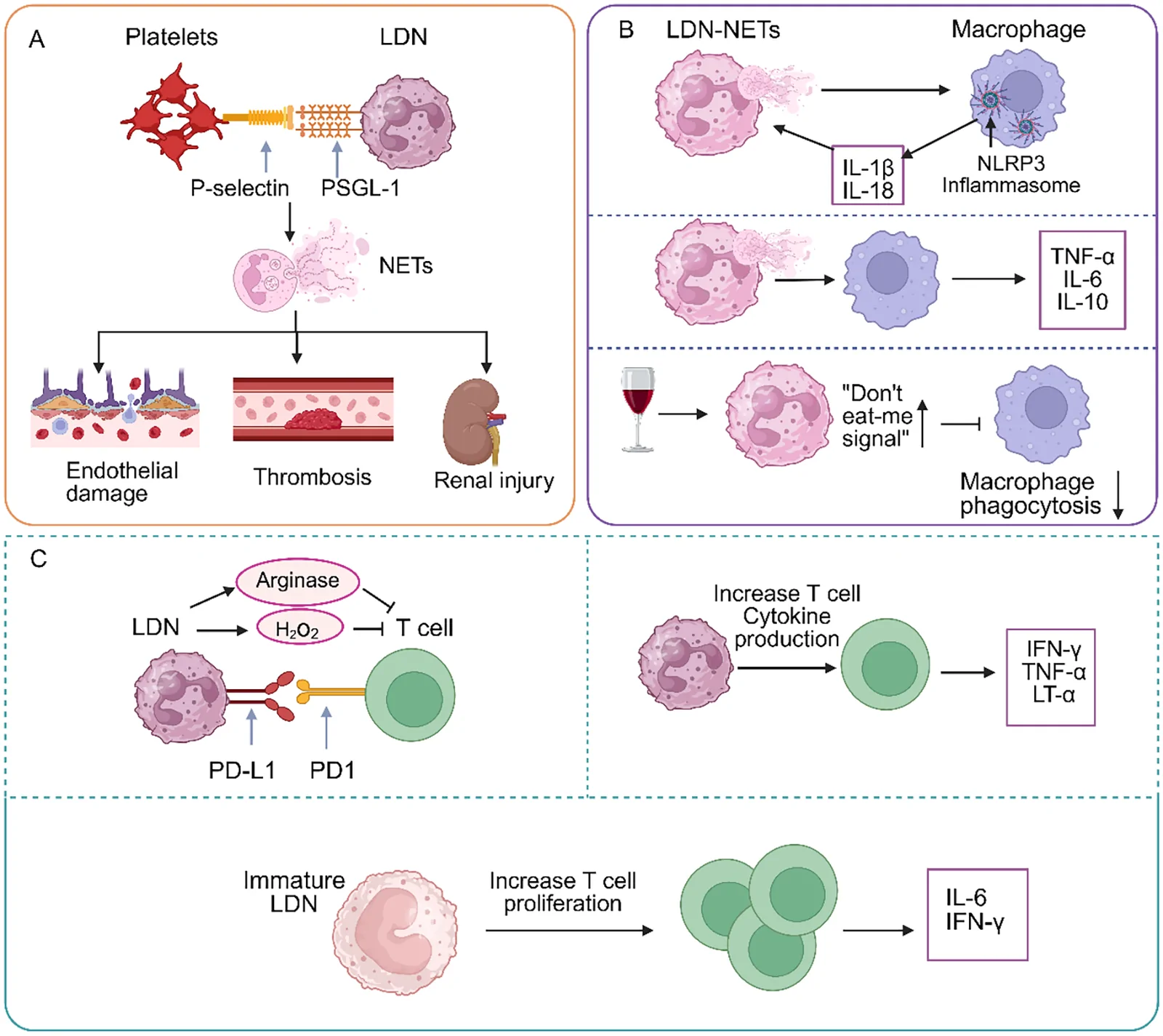
Distinct effector pathways whereby LDNs mediate tissue injury and immune dysregulation in rheumatic autoimmunity. LDNs mediate immune dysregulation and multi-tissue injury via multiple layered effector pathways, as visualized in **Figure 5**. **(A)** Illustrates platelet-LDN adhesion via the P-selectin/PSGL-1 axis, which induces NETosis to cause endothelial impairment, thrombosis and renal pathology. **(B)** Summarizes NET-driven macrophage reprogramming: NETs activate macrophage NLRP3 inflammasome for IL-1β/IL-18 release, promote macrophage cytokine secretion (TNF-α, IL-6, IL-10), and raise “don’t eat-me” signals to reduce macrophage phagocytosis. **(C)** Depicts multifaceted LDN-T cell interactions: LDN suppress T cells through arginase, H_2_O_2_ and PD-L1/PD1 signaling; meanwhile mature and immature LDN subsets enhance T-cell cytokine generation or proliferation, leading to augmented IL-6 and IFN-γ production. Altogether these cascades underpin the broad pathogenic roles of LDNs across multiple rheumatic autoimmune conditions. **Figure 5** Schematic diagram created with https://www.BioRender.com.

Causal-inference framework. We distinguish three levels of evidence throughout this section: (i) genetically anchored, mechanistically proximal evidence in monogenic disorders; (ii) mechanistic evidence from patient-derived cells, tissues or disease models that supports contribution but not necessarily disease initiation; and (iii) observational associations between circulating LDN abundance and clinical activity in polygenic diseases. For common autoimmune diseases, the third category remains dominant, so increased LDN numbers should not be interpreted as proof that LDNs are the primary instigators of disease.

### 3.1 Systemic lupus erythematosus

Systemic lupus erythematosus (SLE), as the prototypic autoimmune disease, has long had its pathogenesis attributed to aberrant activation of adaptive immunity — B-cell production of myriad autoantibodies, immune complex deposition and T-cell dysfunction. Over the past two decades, however, the discovery and in-depth characterization of an abnormal subset of innate immune cells — low-density neutrophils (LDNs), also termed low-density granulocytes (LDGs) — have fundamentally reshaped our conceptual framework of SLE immunopathology. LDNs were first observed in 1986 by Hacbarth and Kajdacsy-Balla in the peripheral blood mononuclear cell (PBMC) fraction of patients with SLE, rheumatoid arthritis (RA) and acute rheumatic fever, but were then dismissed as “contaminating cells” from density gradient centrifugation and ignored (3). It was not until around 2010 that systematic studies, represented by the work of the Kaplan group, confirmed that LDNs enriched in SLE peripheral blood are not experimental artifacts but a heterogeneous disease-associated neutrophil compartment with distinct phenotypic and functional features. Patient-derived studies demonstrated endothelial-injuring and interferon-associated activities, supporting a potential amplifying role for LDN states in SLE immunopathology (20, 74).

#### 3.1.1 Phenotypic heterogeneity and molecularly defined LDN states in SLE

A major methodological limitation is that LDNs are still defined primarily by recovery in the PBMC layer after Ficoll density-gradient centrifugation rather than by unified molecular markers. This separation-based definition inherently encompasses a heterogeneous cell population, rendering direct comparison of results across laboratories difficult and impeding clinical translation. Phenotypically, SLE-derived LDNs express classical myeloid markers CD15, CD66b and CD11b, but their nuclear morphology is heterogeneous, encompassing both segmented mature neutrophils and immature forms such as band cells and metamyelocytes (7, 75). Transcriptomic and epigenomic studies have further revealed that SLE LDNs display global gene expression differences from autologous normal-density neutrophils (NDNs), with significant upregulation of interferon-stimulated genes (ISGs) accompanied by DNA hypomethylation remodeling, suggesting that they are not merely activated NDNs but have undergone a distinct differentiation and reprogramming trajectory (7, 76). A recent transcriptomic study further identified dysregulation of U12-type splicing as a novel molecular feature of lupus neutrophils (77), expanding the repertoire of disease-associated neutrophil states beyond density-based classification.

In recent years, the development of single-cell technologies and multi-omics has propelled the exploration of LDN subset classification. Martin et al. identified a CD98-high LDN subset with metabolic flexibility and enhanced pro-inflammatory potential; as an amino acid transporter and integrin-associated protein, CD98 endows LDNs with greater tissue infiltration and effector capacity through the regulation of cellular metabolism and adhesion (36). Xu et al. found that a CD177-high LDN subset is closely associated with endothelial dysfunction in SLE and contributes to vascular complications via NET-mediated endothelial damage (37). Furthermore, a recent study by Tobío-Parada et al. described a distinctive LDN phenotype associated with subclinical vascular alterations, further confirming the existence of functionally and pathogenically diverse subsets within LDNs (38). Together, these findings indicate that grouping all low-density neutrophils under a single label can obscure clinically relevant functional heterogeneity. The field must transition from a “density-based definition” to a “molecular definition” and establish a unified classification system based on surface markers, transcriptional signatures and functional states; otherwise, conceptual ambiguity will persist.

#### 3.1.2 LDN generation and regulation: platelet–neutrophil interactions and microenvironmental drivers

The cellular origin and molecular mechanisms of LDN expansion in SLE constitute another core controversy. Currently, three main hypotheses exist: abnormal bone marrow granulopoiesis with release of immature cells; phenotypic conversion of peripheral mature neutrophils induced by inflammatory stimuli; and platelet adhesion causing a decrease in neutrophil density. The early view favored a bone marrow origin, positing that IFN-I and granulocyte colony-stimulating factor (G-CSF) in SLE drive emergency granulopoiesis, leading to the premature release of immature neutrophils into the blood (24, 25). However, proteomic and biomechanical analyses show that SLE LDNs differ from normal immature neutrophils in their cytoskeleton, granule protein composition and mechanical properties, supporting their identity as peripherally reprogrammed products (41).

The formation of platelet–neutrophil complexes (PNCs) represents an important mechanistic breakthrough in explaining LDN generation. A 2024 study by Tay et al. demonstrated that platelet TLR7 signaling is essential for PNC formation and LDN generation in lupus nephritis (LN); platelets sense self-nucleic acid ligands via TLR7, become activated, bind to neutrophils, alter their density and functional phenotype, and promote their migration into the low-density layer (39). This discovery connects three major pathological features of SLE — platelet activation, neutrophil abnormalities and the IFN-I pathway — and explains why LDN proportions are highly correlated with disease activity, especially renal involvement. Additionally, fibroblasts in skin lesions can promote the conversion of neutrophils to an LDN phenotype through IL-1β-mediated paracrine signaling, suggesting that the local tissue microenvironment also participates in LDN induction and maintenance (40). Endotoxaemia can also induce elevated LDNs and NETs in pediatric SLE patients, implying that intestinal barrier leakage and translocation of microbial products may be upstream factors driving LDN expansion (78).

#### 3.1.3 SLE-specific extensions of shared LDN effector pathways

Subset-specific LDN signatures and downstream immune cascades dominate the immunopathological progression of SLE (**Figure 6**).

**Figure 6:**
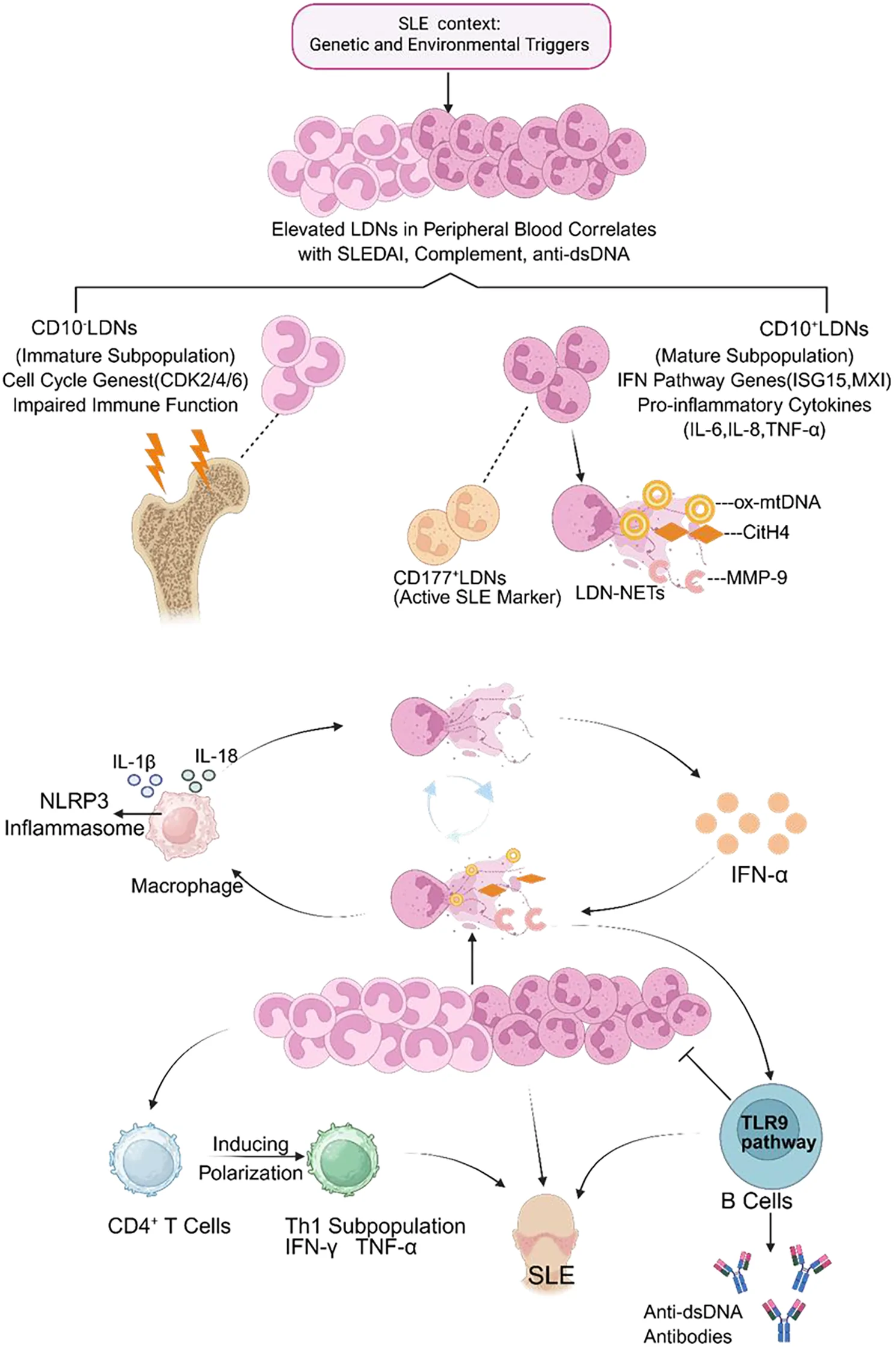
Subset-specific LDN signatures and downstream immune cascades that propagate disease activity in SLE. Under SLE-related genetic and environmental triggers, peripheral LDN counts increase and correlate with SLEDAI, complement alterations and anti-dsDNA levels. CD10^-^ immature LDNs display elevated CDK2/4/6 expression and impaired immune function in bone marrow, while CD10^+^ mature LDNs possess active IFN-responsive gene programs (ISG15, MXI) and abundant pro-inflammatory cytokine outputs. CD177^+^ LDNs denote active SLE, and mature LDN secrete NETs containing ox-mtDNA, CitH4 and MMP-9. Downstream, LDN-derived NETs and IFN-α activate macrophage NLRP3 inflammasome, drive CD4^+^ T-cell polarization toward Th1 cells, and stimulate B-cell TLR9 signaling to produce anti-dsDNA autoantibodies. These coordinated events collectively exacerbate SLE immunopathology. **Figure 6** Schematic diagram created with BioRender.com.

The shared NET–type I interferon pathway is summarized in Section 1.3 and is not repeated here in full. In SLE, the most disease-informative extensions of this pathway are the enrichment of oxidized mitochondrial DNA in LDN-derived NETs, TLR9-dependent pDC activation, impaired NET clearance and GSDMD-associated mitochondrial nucleic-acid release (29, 42–47). These mechanisms are supported by patient-derived cellular studies and are biologically coherent with the SLE interferon signature, but the relative contribution of LDNs versus other neutrophil states to whole-patient IFN activity remains incompletely resolved (48). LDN-mediated T-cell and B-cell interactions likewise support an amplifying role in adaptive autoimmunity without proving that LDNs initiate SLE (24, 49, 50).

##### 3.1.3.1 Vascular endothelial injury and cardiovascular complications

SLE patients face a risk of premature atherosclerosis and cardiovascular disease (CVD) that far exceeds that of the general population, a risk not fully explained by traditional risk factors. LDNs and the NETs they release are implicated as effector cells in vascular injury in SLE. A classic study by Villanueva et al. demonstrated that SLE LDNs can directly damage endothelial cells, disrupt vascular barrier integrity, and infiltrate target organs such as the kidney to cause tissue damage (43). Carmona-Rivera et al. further elucidated the molecular mechanism: NETs activate matrix metalloproteinase-2 (MMP-2), which degrades endothelial cell basement membrane and tight junction proteins, leading to endothelial dysfunction (51). In addition, the CD177^+^ LDN subset participates in the development of SLE vascular complications through NET-mediated endothelial injury (37).

Longitudinal clinical studies support the value of LDNs as CVD predictive biomarkers. A 6-year prospective cohort study by Suárez et al. showed that circulating LDN levels and free DNA can predict the occurrence of CVD and bone mass loss in SLE patients (52). Tobío-Parada et al. also found that specific LDN phenotypes are associated with subclinical vascular changes (38). These lines of evidence indicate that LDNs are not only markers of systemic inflammation in SLE but also potential contributors to vascular damage, supporting a possible link connecting chronic inflammation to premature atherosclerosis in SLE (30, 31).

##### 3.1.3.2 Role in lupus nephritis

The kidney is the most frequently involved target organ in SLE, and LN is a major predictor of poor prognosis. LDNs play multiple roles in the development and progression of LN. First, circulating LDN proportions are positively correlated with renal disease activity, and LDN levels in peripheral blood are significantly elevated in patients with proliferative LN (39, 53). Second, through PNC formation and upregulation of adhesion molecules, LDNs more readily traverse the glomerular endothelial barrier, infiltrate the renal interstitium and glomeruli, and locally release NETs and granular enzymes, causing renal tissue damage and exacerbating inflammatory infiltration (43, 53). Third, NET-derived autoantigens and nucleic acids form immune complexes *in situ* in the kidney, activating the complement cascade and inducing or aggravating glomerulonephritis (44, 53).

In pediatric SLE, LDNs and NETs are also closely associated with disease activity and renal involvement. Saisorn et al. found abundant extracellular traps in the PBMC fraction of pediatric SLE patients, and that endotoxaemia can further induce LDNs and NETs, suggesting similar pathological mechanisms exist in childhood SLE (78, 79). Moreover, neutrophil-associated biomarkers in urine correlate with the degree of renal histopathological injury, offering a potential means for non-invasive monitoring of LN activity (80). Tissue-specific LDN pathogenic mechanisms underlie the multi-organ complications characteristic of SLE (**Figure 7**).

**Figure 7:**
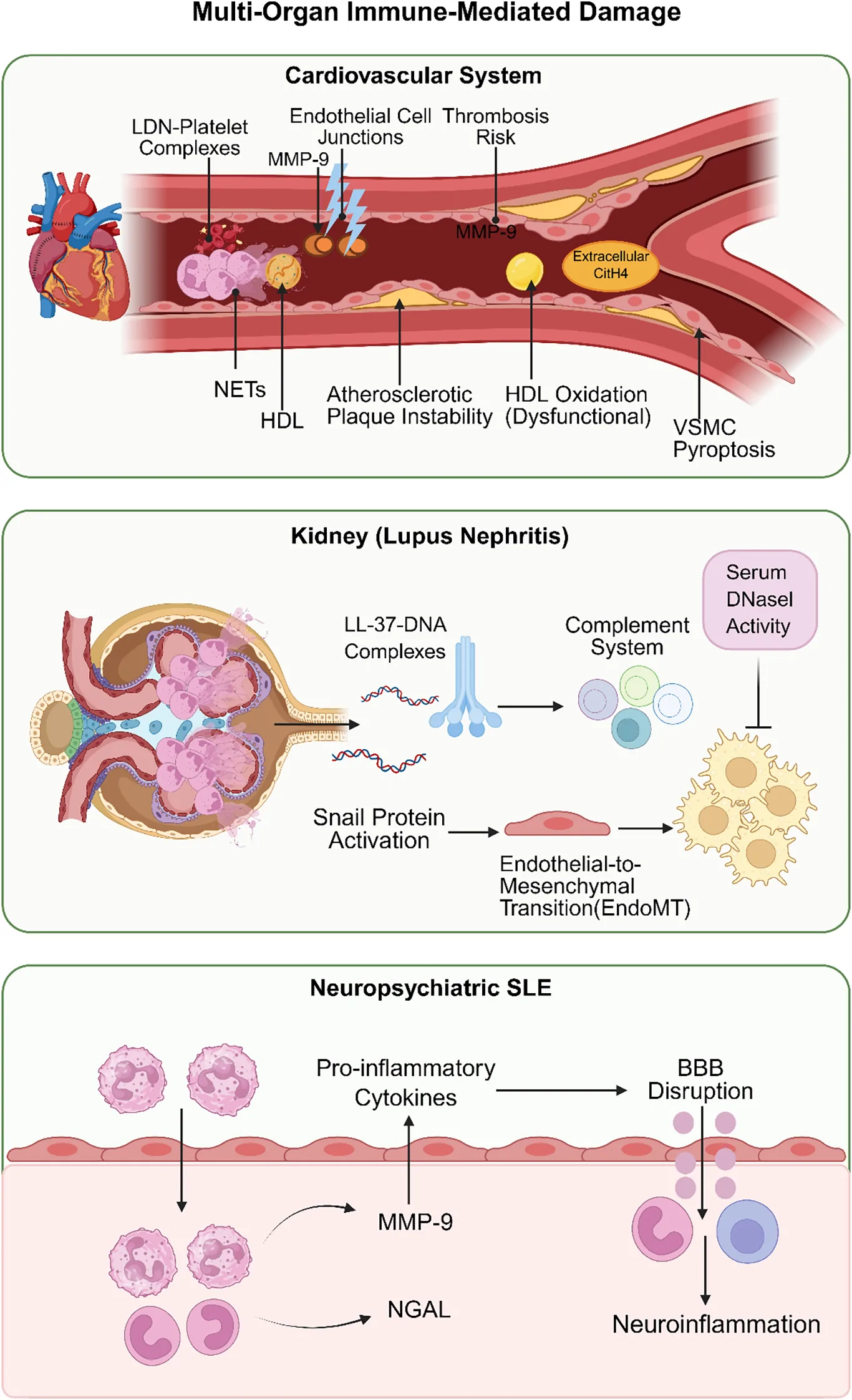
Tissue-specific LDN pathogenic pathways responsible for multi-organ damage in SLE. In the cardiovascular compartment, LDN-platelet complexes release NETs and MMP-9, damaging endothelial junctions, destabilizing atherosclerotic plaques, oxidizing HDL, triggering VSMC pyroptosis and elevating thrombotic risk. Within kidneys affected by lupus nephritis, LDN-derived LL37-DNA complexes activate complement pathways; Snail-mediated EndoMT promotes renal fibrosis, while low serum DNase activity hampers DNA clearance and worsens glomerular injury. For neuropsychiatric SLE, LDN-secreted MMP-9, NGAL and pro-inflammatory cytokines disrupt BBB integrity, thereby triggering central neuroinflammation. These compartmentalized mechanisms jointly produce widespread multi-organ complications in SLE. **Figure 7** Schematic diagram created with BioRender.com.

#### 3.1.4 Clinical associations and therapeutic implications

The association between LDNs and SLE clinical phenotypes has been extensively validated. Multiple studies have confirmed that peripheral blood LDN proportions are positively correlated with SLEDAI scores, rising during active disease and falling upon treatment-induced remission, and can thus serve as potential biomarkers for assessing disease activity (81, 82). Notably, LDNs and NETs are already elevated in patients with incomplete SLE (iSLE), suggesting that neutrophil abnormalities may occur early in the disease, possibly even preceding clinical diagnosis (83). This finding carries important early-warning value — LDNs may become early markers for identifying high-risk populations and predicting disease progression. These associations support clinical relevance but do not establish that LDN expansion precedes or initiates SLE; the same inflammatory cytokine milieu may simultaneously increase disease activity and alter neutrophil density.

In pregnancy-related SLE, LDNs also display clinical relevance. Studies have found that activated LDN levels are elevated in the peripheral blood and intervillous space blood of pregnant SLE patients and are associated with shortened gestation, suggesting that neutrophil abnormalities may participate in the pathological processes underlying adverse pregnancy outcomes in SLE (84, 85). This provides a new perspective for understanding the immune mechanisms of poor pregnancy outcomes in SLE.

In terms of therapeutic translation, targeting LDNs and their effector pathways has shown promise. JAK inhibitors such as tofacitinib can ameliorate neutrophil abnormalities and vascular endothelial function in SLE patients by inhibiting downstream signaling of the IFN pathway, and their efficacy is associated with decreased LDN proportions and reduced NETs (86). Strategies such as inhibiting NET formation, promoting NET degradation and blocking the platelet TLR7 pathway have also shown therapeutic potential in preclinical studies (39, 87). However, a key unresolved question is whether simply depleting LDNs is safe and effective. Given the indispensable role of neutrophils in anti-infective defense, specifically targeting pathogenic LDN subsets or their disease-causing effector molecules, rather than all neutrophils, is likely to be a superior strategy.

### 3.2 Inflammatory arthritis

Inflammatory arthritis encompasses a group of chronic rheumatic diseases characterized by synovial inflammation, bone erosion and immune dysregulation, including rheumatoid arthritis (RA), juvenile idiopathic arthritis (JIA), gouty arthritis, psoriatic arthritis (PsA) and ankylosing spondylitis (AS), among other phenotypes. Historically, neutrophils were regarded as “terminal effector cells” of joint inflammation, mediating tissue damage solely through the release of proteases and reactive oxygen species. The discovery and characterization of low-density neutrophils (LDNs) — have thoroughly rewritten this conceptual framework. This cell subset, named for its co-sedimentation with the peripheral blood mononuclear cell layer during Ficoll density gradient centrifugation, was first described by Hacbarth et al. in 1986 in the peripheral blood of patients with RA, SLE and acute rheumatic fever (3), but only in the past decade, with the development of multi-omics technologies and functional immunology, has its central regulatory role in the pathogenesis of inflammatory arthritis been systematically revealed. This section critically examines the phenotypic heterogeneity, pathogenic mechanisms and clinical translational value of LDNs in major forms of inflammatory arthritis, focusing on current consensus, controversies and unresolved scientific questions.

#### 3.2.1 Phenotypic characteristics and disease associations of LDNs in different inflammatory arthritides

##### 3.2.1.1 Rheumatoid arthritis: functional specialization of a pathogenic subset

RA is the most intensively studied inflammatory arthritis model for LDNs. A landmark 2017 study by Wright et al. was the first to demonstrate, through transcriptomics, that RA LDNs exhibit fundamental gene expression differences from normal-density neutrophils (NDNs), rather than representing merely a different activation state (35). RA-LDNs display a markedly immature phenotype, with band-shaped or hyposegmented nuclear morphology, reduced surface expression of CD10, CD16 and CXCR2, and a signaling defect in response to TNF-α stimulation — their TNF-induced respiratory burst and degranulation responses are significantly weaker than those of NDNs, suggesting that this subset is not a classical “activated neutrophil” but a pathogenic subset with a unique transcriptional regulatory program (35). This discovery overturned the traditional view of neutrophil homogeneity and provided a new perspective for understanding neutrophil dysfunction in RA.

Clinically, peripheral blood LDN proportions correlate positively with RA disease activity (DAS28), erythrocyte sedimentation rate and C-reactive protein levels, and LDNs heavily infiltrate inflamed synovial tissue (54). Notably, after leukocytapheresis treatment, the proportion of immature neutrophils in RA peripheral blood decreases in parallel with clinical improvement, indirectly supporting the potential of LDNs as a biomarker of treatment response (88). However, current studies have significant methodological limitations: most rely solely on density gradient centrifugation to isolate LDNs, lacking precise subset classification based on single-cell transcriptomics, and therefore cannot distinguish “true” immature neutrophils from activated mature neutrophils that have decreased in density. This is a core reason for the widely discrepant LDN proportions reported across different studies.

##### 3.2.1.2 Juvenile idiopathic arthritis: a marker of innate immune dysregulation in childhood arthritis

JIA, the most common rheumatic disease of childhood, has seen its LDN research start relatively late but progress rapidly. As early as 2003, Ronchezel et al. observed that the neutrophil contamination rate in the PBMC fraction of JIA patients was significantly elevated and correlated with disease activity (55). Ramanathan et al. identified a prominent neutrophil activation signature in JIA patients through gene expression profiling in 2018, and confirmed that this signature primarily originates from circulating LDNs, proposing that an LDN-associated gene signature could serve as a non-invasive biomarker for JIA (56). A 2023 study by Parackova et al. further systematically characterized the immunophenotype of JIA-LDNs, revealing upregulation of CD11b, CD66b and the activation marker CD64, with downregulation of the maturation marker CD10, and functionally exhibiting enhanced pro-inflammatory cytokine secretion and delayed apoptosis (57).

Critically examining the JIA field reveals three major gaps: first, the phenotypic and functional heterogeneity of LDNs across different JIA subtypes (systemic, polyarticular, oligoarticular) remains unelucidated; second, given that the pediatric immune system is still developing, it is unknown whether LDN expansion reflects developmental abnormality or is purely inflammation-driven; and third, a causal relationship between LDNs and the production of JIA-specific autoantibodies (e.g., antinuclear antibodies) has not been established. Systemic JIA is clinically highly similar to adult-onset Still’s disease (AOSD), which is already known to feature prominent LDN expansion positively correlated with serum G-CSF levels (89, 90); this connection warrants further validation in JIA research.

##### 3.2.1.3 Crystal-induced arthritis: density transition as a critical step in inflammation amplification

Gouty arthritis is triggered by the deposition of monosodium urate (MSU) crystals, and its acute attacks are characterized by massive neutrophil infiltration. A 2020 study by Tang et al. first revealed that MSU crystals can directly induce a “density transition” in normal-density neutrophils, converting them into an LDN phenotype accompanied by reduced forward scatter, elevated side scatter and upregulation of activation markers (91). This finding provides a new explanation for the rapid amplification mechanism of acute gout attacks: neutrophils recruited to the joint are stimulated by MSU, decrease in density, and may alter their adhesive properties and migratory capacity, thereby accelerating the inflammatory cascade.

Nevertheless, LDN research in crystal-induced arthritis is still in its infancy. Early studies found that low-density lipoprotein (LDL) can inhibit neutrophil activation by binding MSU crystals (92, 93), but the regulatory effect of LDL on LDN formation is completely unknown. Furthermore, whether a similar LDN conversion mechanism exists in other crystal-induced arthritides, such as calcium pyrophosphate deposition disease (CPPD), lacks research evidence. A core challenge in this field is determining whether the density change of neutrophils during acute gout attacks is the result of functional subset differentiation or merely a secondary phenomenon caused by cell activation and degranulation; distinguishing between these two possibilities is crucial for judging their value as therapeutic targets.

##### 3.2.1.4 Spondyloarthritis spectrum: neglected participants from innate immunity

Compared with RA and SLE, LDN research in PsA and AS is exceedingly scarce. Teague et al. found in psoriasis research that low-density neutrophils interacting with platelets can promote vascular inflammation and atherosclerosis (61); since PsA is the articular manifestation of psoriasis, similar mechanisms are presumed to exist. Okyar et al. reported in 2024 that the proportion of immature granulocytes (IG) is elevated in the peripheral blood of AS patients and that IG levels decrease after biologic therapy (94); although LDNs were not directly identified, immature granulocytes are an important component of the LDN pool. The IL-23/IL-17 axis is the core pathogenic mechanism in spondyloarthritis, and LDNs have been shown to secrete IL-17 and promote Th17 differentiation; the functional link between the two is an extremely valuable research direction.

#### 3.2.2 Arthritis-specific mechanisms and mechanistic uncertainties

The generic NET–IFN-I pathway is described in Section 1.3. In rheumatoid arthritis, the more disease-specific issue is the coupling of NET-associated citrullinated proteins to anti-citrullinated protein antibody (ACPA) biology and synovial inflammation. LDN-derived or neutrophil-derived NETs can provide citrullinated autoantigens and activate synovial stromal cells, supporting an amplifying loop between neutrophils, autoantibodies and the synovium (54). However, ACPAs often precede clinical arthritis by years, whereas reported LDN expansion usually tracks active inflammation; this temporal mismatch argues against assuming that circulating LDN expansion is the initiating event.

Platelet–neutrophil interactions are another plausible arthritis-specific modifier, but direct evidence remains limited. Platelet adhesion and P-selectin/PSGL-1 signaling can alter neutrophil activation and density, yet much of the mechanistic framework derives from lupus nephritis rather than arthritis (39). Similarly, changes in total neutrophil counts after JAK inhibition do not establish selective effects on LDNs. These observations should therefore be treated as mechanistic hypotheses requiring subset-resolved validation rather than as established LDN-directed mechanisms.

Current arthritis data therefore support a model in which LDNs may amplify joint inflammation in selected patients, while the relative contributions of immature granulopoietic cells, peripherally activated mature neutrophils and tissue-adapted synovial neutrophils remain unresolved. Paired blood–synovium single-cell studies and longitudinal sampling before and after treatment will be necessary to determine whether circulating LDN states are mechanistically linked to the pathogenic neutrophils present in the joint.

#### 3.2.3 Opportunities, controversies and unresolved issues in clinical translation

##### 3.2.3.1 Biomarker potential: advantages and limitations

LDNs and their associated gene signatures hold multiple potential values as biomarkers for inflammatory arthritis: aiding early diagnosis, assessing disease activity, predicting treatment response and judging prognosis. In RA, the correlation between LDN proportions and DAS28 scores is superior to that of total neutrophil counts (49); in JIA, neutrophil gene expression signatures can distinguish active disease from remission (56). However, their clinical translation faces three major obstacles:

1. Lack of standardized detection methods: At present, LDN isolation relies mainly on Ficoll density gradient centrifugation, which is cumbersome and poorly reproducible; isolation protocols vary significantly across laboratories, making it difficult to establish unified reference ranges.
2. Insufficient disease specificity: LDN expansion is observed in various autoimmune diseases, infections and tumors; standalone detection lacks diagnostic specificity and requires combined interpretation with clinical context and other indicators.
3. Unresolved functional heterogeneity: LDNs are not a uniform population but encompass immature precursors, activated mature neutrophils and myeloid-derived suppressor cells; simple enumeration cannot reflect their functional status.

##### 3.2.3.2 Therapeutic target exploration and challenges

Strategies for targeting LDNs to treat inflammatory arthritis can be classified into three categories: reducing LDN generation, inhibiting their effector functions, and promoting their clearance. The effects of existing DMARDs and biologics on LDNs are largely “off-target”, and specific interventions are lacking. For example, tocilizumab treatment in RA can reduce LDN proportions and decrease NETosis while improving endothelial function (95), but whether this is a direct effect of IL-6 receptor blockade or an indirect consequence of inflammation control remains unresolved.

The core dilemma in developing LDN-selective drugs is the “specificity” problem: as neutrophils are the body’s first line of anti-infective defense, wholesale suppression may lead to severe infection risk. An ideal therapeutic target should selectively target pathogenic LDN subsets without impairing normal defensive functions. Currently, investigational targets such as SYK inhibitors, PAD4 inhibitors and CXCR2 antagonists all face similar issues. Although the SYK inhibitor fostamatinib showed potential to restore myeloid homeostasis in COVID-19 (96), its clinical trial results in RA have been disappointing, partly due to insufficient target selectivity.

### 3.3 Psoriasis

Psoriasis is a chronic inflammatory skin disease centered on aberrant activation of the Th17 axis. Its lesional histology has long been defined by epidermal hyperplasia, parakeratosis and neutrophilic infiltration in the dermal papillae, with Munro microabscesses and Kogoj spongiform pustules as hallmark pathological changes (97, 98). However, traditional studies largely treated neutrophils as a homogeneous effector population. It was not until the discovery of the low-density neutrophil (LDN) compartment that the deeper significance of neutrophil heterogeneity in psoriasis pathogenesis was revealed (13). Recent studies indicate that LDNs are not only numerically correlated with disease activity but also deeply involved in the initiation, progression and comorbidities of psoriasis through multiple mechanisms, including preferential skin infiltration, enhanced release of neutrophil extracellular traps (NETs), mediation of vascular endothelial injury and modulation of treatment responses.

#### 3.3.1 Phenotypic heterogeneity and quantitative abnormalities of circulating LDNs in psoriasis

As early as the era of PBMC isolation by density gradient centrifugation, researchers noticed an abnormal increase in granulocyte-like cells in the PBMC layer of psoriasis patients. A systematic review by Wang et al. was the first to clearly indicate that the proportion of LDNs in the peripheral blood of psoriasis patients is significantly elevated compared with healthy controls, and their abundance shows a positive correlation with disease severity (13). Subsequent flow cytometric characterization revealed that psoriasis-associated LDNs display a CD15^+^CD16^+^CD66b^(high)CD14^(low) myeloid phenotype, accompanied by CD62L downregulation and CD11b upregulation, indicating a pre-activated state. More importantly, this population is not homogeneous but comprises both mature and immature subsets, with absence of CD10 expression serving as a discriminator for immature LDNs (99). This phenotypic heterogeneity directly challenges the simplistic notion that “LDNs are just activated neutrophils” and suggests that both emergency granulopoiesis and peripheral reprogramming may jointly contribute to LDN formation in psoriasis. Multiple clinical studies have also confirmed that the neutrophil-to-lymphocyte ratio (NLR) in peripheral blood is significantly elevated in psoriasis and psoriatic arthritis patients and can serve as a potential marker reflecting disease activity (100, 101).

The lineage characteristics of LDNs differ significantly across clinical subtypes of psoriasis. A study by Yu et al. focusing on acute generalized pustular psoriasis (GPP) identified a unique population of immature LDNs in the peripheral blood, phenotypically and functionally distinct from the mature LDN subset found in psoriasis vulgaris (58). GPP is characterized by aberrant activation of the IL-36 pathway, massive intraepidermal neutrophil aggregation and systemic inflammatory responses; the expansion of immature LDNs echoes IL-36-driven bone marrow hematopoietic stress, providing a cellular basis for understanding the immunopathological divergence between pustular and plaque psoriasis. This finding simultaneously raises a key question: does the difference in LDN subset composition determine the direction of phenotypic conversion in different psoriasis types, or is it merely a secondary consequence of varying inflammation intensity? No longitudinal study has yet been able to provide a causal answer.

#### 3.3.2 Molecular mechanisms of preferential infiltration of LDNs into skin lesions and their pathogenic effects

Circulating LDNs are not merely blood-based inflammatory markers; their active infiltration into skin tissue is the core of their pathogenic role. Using an imiquimod (IMQ)-induced psoriasiform dermatitis mouse model, Dziadowiec et al. provided the first direct evidence through adoptive transfer experiments that low-density neutrophils (LDNeu) possess significantly stronger lesional tropism than conventional neutrophils (cNeu) and can be preferentially recruited to inflamed skin and locally aggregate (59). Mechanistically, upregulation of the chemokine receptor CXCR4 on the LDN surface is an important molecular basis for their directed migration, while high expression of CXCL12 in the skin lesions forms a chemotactic gradient. This finding functionally explains why the neutrophils infiltrating psoriatic lesions more closely resemble circulating LDNs than cNeu.

Within psoriatic lesions, the shared NET pathway acquires disease-specific features. Psoriasis-associated LDNs show increased neutrophil elastase relative to its endogenous inhibitor SLPI, and NET-associated proteases can activate IL-36-family cytokines, linking neutrophil effector activity to the keratinocyte–IL-36 inflammatory circuit (60). These observations distinguish psoriasis from a generic NET-mediated model, although the extent to which the phenotype is intrinsic to circulating LDNs versus induced by the skin cytokine environment remains unresolved.

It is worth critically noting that current studies generally treat the NET-releasing capacity of LDNs as an intrinsic property, but increasing evidence indicates that cytokines within the skin microenvironment (e.g., TNF-α, IL-17A, IL-36γ) can further reprogram the functional phenotype of LDNs. In other words, the pre-activated state of circulating LDNs and the inflammatory reinforcement within the skin lesion may be two independent but synergistic pathogenic layers that together drive the maintenance and recurrence of chronic inflammation in psoriasis.

#### 3.3.3 Pathological mechanisms of LDN-mediated cardiovascular comorbidities in psoriasis

The elevated risk of cardiovascular disease (CVD) in psoriasis patients, often accompanied by metabolic disorders such as dyslipidaemia, is now a consensus, and neutrophils are thought to play an important role (102). LDNs are considered key cellular mediators linking skin inflammation to systemic vascular injury. Using coronary CT angiography, Teague et al. confirmed that circulating LDN levels in psoriasis patients are significantly positively correlated with non-calcified coronary plaque burden, and that the quantity of LDN–platelet aggregates is an independent predictor of coronary plaque instability (61). Functional studies further revealed that LDN–platelet aggregates can induce upregulation of a pro-inflammatory transcriptional profile in endothelial cells, increasing the expression of adhesion molecules and chemokines such as VCAM-1, IL-1β and CXCL10 by 2- to 8-fold, with an intensity of endothelial damage induction approximately 50% higher than that of conventional neutrophils. A review by Sanda et al. systematically elaborated on the pathological pathways through which LDNs promote atherosclerotic plaque progression and thrombosis via NET release, pointing out that the sustained expansion of LDNs in the psoriatic state constitutes the cellular basis of the “skin–vascular” inflammatory axis (103). Furthermore, the pathogenic role of LDNs in musculoskeletal comorbidities such as psoriatic arthritis is receiving increasing attention, with related studies demonstrating that low-density granulocytes can accumulate within joints and participate in inflammatory damage (104).

The generic endothelial and thrombotic effects of NETs are summarized in Section 1.3. In psoriasis, the clinically distinctive observation is the association of circulating LDNs and LDN–platelet aggregates with non-calcified coronary plaque burden and vascular inflammatory phenotypes (61, 103). These data support a potential skin–vascular inflammatory link, but they do not resolve whether LDN expansion is an upstream contributor shared by cutaneous and vascular disease or a downstream consequence of severe systemic inflammation. Existing Mendelian-randomization data on BMI, psoriasis and total neutrophil counts do not establish causality for the LDN compartment itself (105).

#### 3.3.4 LDNs as biomarkers and intervention targets for treatment response in psoriasis

With the advent of the biologic era, the significance of LDNs in psoriasis therapeutics is gradually emerging. Through transcriptomic analysis, Lu et al. discovered that in psoriasis patients treated with guselkumab (an anti-IL-23p19 monoclonal antibody), the baseline gene signature of early fast responders is significantly enriched in LDN-associated gene sets, suggesting that the baseline status of circulating LDNs may predict therapeutic sensitivity to IL-23 pathway blockade (62). Differential gene expression analysis showed that LDN activation-related molecules such as IL-1β, CXCL8, S100A12 and MMP9 are rapidly downregulated after treatment in the fast responder group, while the slow responder group shows the opposite trend. The clinical significance of this finding is that LDNs may serve not only as inflammatory markers but also as predictive biomarkers for stratified treatment, helping to identify patient subgroups suitable for therapies targeting the IL-23/IL-17 axis.

However, existing therapeutic studies have notable methodological limitations. Most observations remain at the level of describing changes in LDN proportions before and after treatment, lacking systematic assessment of functional phenotypic remodeling of LDNs. For instance, although IL-17A inhibitors such as secukinumab can significantly improve skin inflammation and reduce some cardiovascular risk markers, their long-term effects on LDN quantity and function remain unclear (106). Moreover, treatment strategies directly targeting LDNs have not yet entered clinical translation — although traditional drugs like methotrexate (107) and JAK inhibitors (108) may affect neutrophil generation and activation, none are LDN-specific interventions. How to selectively eliminate or reprogram pathogenic LDNs without compromising host defense function is the core challenge for future translational research.

### 3.4 Other rheumatic and autoinflammatory diseases

The abnormal expansion and functional dysregulation of low-density neutrophils (LDNs) are not unique to systemic lupus erythematosus (SLE) but are a shared immunopathological feature across the continuum of autoimmune–autoinflammatory diseases. From multisystem autoimmune diseases (idiopathic inflammatory myopathies), systemic vasculitides (Behçet’s disease, deficiency of adenosine deaminase 2), and spondyloarthritis to monogenic autoinflammatory syndromes (PAPA syndrome, NEMO deficiency), LDNs participate in the initiation, progression and outcome of diseases with varying abundances and functional phenotypes. This section systematically synthesizes the research evidence on LDNs in these conditions, focusing on cross-disease shared pathogenic mechanisms versus disease-specific differences, and critically examines the methodological limitations and translational medicine bottlenecks of current research.

#### 3.4.1 Idiopathic inflammatory myopathies: LDN associations with muscle and extra-muscular involvement

Idiopathic inflammatory myopathies (IIMs) are a heterogeneous group of chronic autoimmune diseases characterized by progressive inflammatory injury to skeletal muscle, frequently accompanied by extra-muscular manifestations such as interstitial lung disease (ILD), skin involvement, arthritis and cardiovascular complications (63, 64). The traditional view holds that the effector cells in myositis are primarily T lymphocytes and macrophages, but recent studies have confirmed that aberrant neutrophil activation, especially LDN expansion, is a long-underestimated key link in IIM immunopathology (65).

Elevated circulating LDN proportions are a prominent immunological feature of active IIM. Torres-Ruiz et al. confirmed in an adult IIM cohort that peripheral blood LDN proportions are positively correlated with disease activity, and that the LDN–NET axis can serve as a potential biomarker of disease activity (66). In the dermatomyositis (DM) subtype, Zhang et al. were the first to discover that LDN levels in the PBMC fraction of patients with ILD are significantly higher than in those without ILD, suggesting that LDNs may participate in the pathological process of pulmonary interstitial injury (67). This finding has important clinical significance — DM-ILD is the leading cause of poor prognosis and death, and LDNs may become a hematological biomarker for early identification of high-risk populations.

Mechanistically, IIM studies largely converge on the shared NET–nucleic acid inflammatory pathways summarized in Section 1.3. Disease-specific observations are the associations of the circulating LDN/NET phenotype with muscle disease activity and interstitial lung disease, whereas direct evidence that a defined LDN subset migrates into muscle and is necessary for myofiber injury remains limited (63–67). Thus, current IIM data support biological plausibility and clinical association more strongly than tissue-level causality.

It is worth critically noting that current LDN research in IIMs has three major limitations: first, most studies are cross-sectional in design and lack longitudinal cohort validation of the causal temporal relationship between LDNs and disease progression; second, the phenotypic heterogeneity of LDNs across different IIM subtypes (DM, PM, inclusion body myositis) has not been systematically dissected, making it impossible to distinguish shared disease features from subtype-specific changes; and third, the evidence for LDN infiltration in muscle tissue is still insufficient — whether circulating LDNs can migrate to inflamed muscle and exert local effects still requires histological evidence (65).

#### 3.4.2 Monogenic autoinflammatory diseases: genetically proximal evidence for neutrophil dysregulation

Monogenic autoinflammatory diseases are caused by defined variants in innate-immune pathways and therefore provide genetically anchored models in which the upstream lesion is known. This offers more proximal causal inference for neutrophil dysregulation than is available in polygenic autoimmune diseases, where circulating LDN measurements are usually cross-sectional or longitudinal associations. Nevertheless, a causal genetic lesion does not automatically prove that the low-density phenotype itself is necessary or sufficient for disease; LDN expansion may be one mediator within a broader mutation-driven inflammatory network.

Deficiency of adenosine deaminase 2 (DADA2) is a paradigm for studying LDN pathogenic mechanisms. DADA2 results from biallelic mutations in the ADA2 gene and manifests as systemic vasculitis, ischaemic stroke and premature atherosclerosis. A landmark study by Carmona-Rivera et al. confirmed that circulating LDNs are significantly expanded in patients with active DADA2 and possess an enhanced capacity for spontaneous NET formation; aberrant adenosine metabolism is the core driver — accumulated adenosine activates neutrophils via A1 and A3 adenosine receptors, inducing NETosis through reactive oxygen species (ROS)- and peptidylarginine deiminase 4 (PAD4)-dependent pathways (68). More importantly, DADA2-derived NETs can polarize macrophages toward a pro-inflammatory M1 phenotype, inducing robust TNF-α production and forming a positive-feedback inflammatory loop of “adenosine accumulation–LDN expansion–NETosis–TNF release”. After anti-TNF therapy induces clinical remission, circulating LDN proportions and NET levels decrease in parallel, supporting their potential as pharmacodynamic monitoring markers; this treatment-associated normalization does not by itself prove that LDNs are the indispensable causal intermediate (68).

PAPA syndrome (pyogenic arthritis, pyoderma gangrenosum, acne) is caused by mutations in the PSTPIP1 gene and is characterized by excessive IL-1β production. Mistry et al. found that PAPA patients not only have elevated circulating LDNs and enhanced NET-forming capacity but, more critically, exhibit a defect in serum NET degradation capacity, leading to a prolonged tissue half-life of NETs and sustained pro-inflammatory effects (69). Mechanistically, IL-1β can directly induce neutrophil NET formation, and the IL-1 receptor antagonist anakinra can reverse this phenotype; conversely, PAPA-derived NETs can stimulate macrophages to release IL-6 and IL-1β, further amplifying inflammation. The detection of an LDN-characteristic gene expression profile in patient skin biopsies confirms that circulating LDNs can migrate to inflamed tissue and participate in pathological injury (69).

NEMO deficiency syndrome (NF-κB essential modulator deficiency) results from IKBKG gene mutations leading to aberrant NF-κB signaling, manifesting as immunodeficiency combined with autoinflammation. Surucu Yilmaz et al. confirmed that episodes of autoinflammation in patients are accompanied by significant LDN expansion and neutrophil dysfunction, and that LDN levels normalize after hematopoietic stem cell transplantation (HSCT), supporting an intrinsic hematopoietic contribution to the neutrophil phenotype; however, the observation does not establish that low density itself is the disease-causing property (70).

Across these monogenic disorders, defined upstream mutations, mechanistic neutrophil abnormalities and treatment- or transplantation-associated phenotype normalization together provide comparatively strong, genetically proximal evidence that neutrophil dysregulation participates in disease biology (68–70). The causal claim should nevertheless be formulated at the level of the neutrophil/NET inflammatory axis rather than the density phenotype alone. By contrast, in SLE, RA, JIA, psoriasis and most other polygenic disorders, evidence is dominated by correlations between LDN abundance and disease activity, organ involvement or treatment response. Such associations are compatible with at least three models—LDNs as drivers, LDNs as inflammation-induced amplifiers, or LDNs as biomarkers/bystanders of emergency granulopoiesis—and current human data rarely distinguish among them.

#### 3.4.3 Behçet’s disease: numerical expansion without consistent functional alteration

Behçet’s disease (BD) is a systemic vasculitis classically characterized by neutrophil hyperfunction, with excessive neutrophil activation considered an important component of the pathophysiology of mucosal ulcers, ocular inflammation and vasculitis. Based on the pathogenic role of LDNs in other vasculitides, it was hypothesized that LDN proportions would be elevated in BD patients with enhanced function, representing the main carrier of abnormal neutrophil activation.

However, a study by Murad et al. reached a paradoxical conclusion: although the proportion of peripheral blood low-density neutrophils (LDNs) was indeed significantly higher in BD patients than in healthy controls, the LDNs did not significantly differ from normal-density neutrophils (NDNs) in phagocytic function, ROS production, degranulation or NET-forming capacity, and could not explain the overall neutrophil hyperfunction in BD (71). This “quantity–function dissociation” phenomenon challenges the traditional notion that LDNs are uniformly pathogenic.

Several interpretations can be offered for this finding: first, LDNs in BD may be predominantly immunoregulatory or immature subsets rather than pathogenic ones, and their expansion could represent a compensatory response to chronic inflammation; second, the functional abnormality of neutrophils in BD may primarily occur in the normal-density subset, with LDNs not being the main effector cells; and third, methodological heterogeneity may be a key factor — different studies employ different density gradient separation protocols and flow cytometric gating strategies, leading to discrepancies in the definition and purity of LDNs, directly affecting the reliability of functional comparisons (2).

The case of BD serves as a caution: LDNs cannot simply be regarded as a uniformly pathogenic cell population; their functional phenotype is highly disease-specific. Without first clarifying disease-specific LDN subset characteristics, generalizing LDNs as “pathogenic subsets” risks over-extrapolation.

#### 3.4.4 Spondyloarthritis: limited evidence and cardiovascular hypotheses

Spondyloarthritis (SpA), including ankylosing spondylitis (AS), is characterized by axial inflammation and enthesitis and is associated with increased cardiovascular risk (72, 73). Although neutrophil activation and NET-related pathways have been implicated in SpA, direct evidence defining a circulating LDN compartment, its phenotype or its clinical significance remains scarce. It is therefore premature to infer an LDN-mediated vascular mechanism from general neutrophil or psoriasis data; at present, LDN involvement in SpA should be regarded as a testable hypothesis rather than an established component of pathogenesis.

It must be emphasized that this area is currently at the hypothesis stage, lacking direct clinical validation. Future research must answer three core questions: Are circulating LDNs truly elevated in SpA patients? Are LDN levels correlated with disease activity, radiographic progression and cardiovascular events? Can targeting the LDN–NET axis improve inflammatory control and cardiovascular outcomes in SpA? Filling these gaps will greatly expand the research landscape of LDNs in rheumatic diseases. Cross-disease differences in LDN characteristics, clinical significance, and evidence limitations are summarized in **Table 2**.

**Table 2:** Cross-disease comparison of LDN characteristics, disease-specific findings, clinical significance and evidence limitations.

Disease/context	Reported LDN features	Disease-specific observations	Clinical significance	Major evidence limitation
SLE/LN	Mixed immature and mature/activated states; CD98-high and CD177-high phenotypes	Platelet TLR7–neutrophil complexes, ox-mtDNA/IFN axis, endothelial and renal injury (29, 36–39, 41–53)	Activity, vascular risk and renal involvement associations	Most human data remain observational; subset causality is not established
RA	Frequently immature/low-CD10 phenotype with distinct transcriptional program (35)	Synovial infiltration; association with DAS28 and inflammatory markers (54)	Candidate activity/treatment-response biomarker	Density-based isolation and limited subset-resolved longitudinal data
JIA	Activated/immature phenotype with CD11b/CD66b/CD64 changes (55–57)	Prominent neutrophil/LDN gene signatures in childhood arthritis	Potential activity-state marker	Small cohorts; limited causal and tissue-level evidence
Psoriasis/GPP	Activated mature LDNs in plaque disease; immature LDN expansion in GPP (58)	Skin tropism, CXCR4/CXCL12 recruitment, NE–SLPI/IL-36 coupling (59, 60)	Associations with severity, vascular phenotype and treatment response (61, 62)	Unclear intrinsic versus microenvironment-induced phenotype
IIM	Expanded circulating LDN/NET phenotype	Associations with activity and interstitial lung disease (63–67)	Candidate disease-activity and risk marker	Cross-sectional evidence; tissue infiltration/necessity not established
Monogenic autoinflammatory disease	LDN expansion and neutrophil dysfunction downstream of defined mutations	DADA2, PAPA and NEMO deficiency provide genetically proximal mechanistic evidence (68–70)	Mechanistic model systems and potential pharmacodynamic markers	Low-density phenotype itself is not proven necessary or sufficient
Behçet disease/SpA	BD: numerical expansion without consistent functional difference; SpA: direct LDN evidence scarce (2, 71–73)	Demonstrates disease-specific heterogeneity and important negative/uncertain evidence	Primarily hypothesis-generating	Limited cohorts and lack of validated LDN-specific endpoints

## 4 Therapeutic implications of low-density neutrophil biology in rheumatic disease

The therapeutic relevance of LDN biology lies in the possibility that disease-associated neutrophil states or their effector pathways may be modifiable without broadly suppressing host-protective neutrophil functions. This concept remains attractive but incompletely validated. Most current anti-rheumatic therapies were not developed to target LDNs, and many proposed LDN-directed strategies are supported only by preclinical data or by extrapolation from general neutrophil and tumor/MDSC biology.

Accordingly, this section treats LDNs as a heterogeneous, density-defined compartment rather than a uniformly pathogenic cell population. Low-density neutrophils are detectable in healthy individuals (8), and their functions vary with maturation state, tissue context and inflammatory environment. The therapeutic objective should therefore be selective modulation of disease-associated pathogenic states or effector pathways, not indiscriminate elimination of all cells recovered in the low-density fraction.

### 4.1 Therapeutic rationale and evidentiary framework

Three evidence levels should be distinguished. First, a limited number of studies in patients with rheumatic disease show that effective anti-inflammatory therapy can alter LDN abundance, NET formation or related vascular phenotypes. Second, mechanistic studies using patient-derived cells and rheumatic-disease models identify potentially druggable pathways, but do not establish clinical efficacy. Third, a substantial number of proposed strategies derive from general neutrophil biology, cancer-associated PMN-MDSCs or other inflammatory diseases and should be considered hypothesis-generating until validated in rheumatic disease.

Direct human evidence remains limited and largely demonstrates indirect modulation rather than LDN-specific pharmacology. In SLE, a phase 1 trial of tofacitinib reported improvement in vascular and neutrophil-related abnormalities (86), while in RA, tocilizumab treatment was associated with reduced LDN proportions and NETosis together with improved endothelial function (95). Although JAK inhibitors (baricitinib, tofacitinib) are approved for RA (109–112), their selective effects on the LDN compartment have not been systematically characterized in RA cohorts. These agents target JAK or IL-6 signaling rather than LDNs themselves, and current data cannot determine whether changes in the low-density compartment are a cause of clinical benefit or a downstream consequence of inflammation control.

Disease-proximal mechanistic studies provide stronger support for selected pathways than for direct cell depletion. Examples include platelet TLR7-dependent formation of platelet-neutrophil complexes and LDNs in lupus nephritis (39), interferogenic oxidized mitochondrial DNA in SLE NETs (29), and disease-associated LDN phenotypes linked to endothelial dysfunction (37). These findings identify candidate intervention nodes, but pathway involvement should not be equated with demonstrated therapeutic efficacy.

The evidence hierarchy for candidate LDN-related therapeutic strategies in rheumatic disease is summarized in **Table 3** and is used below to separate clinically observed effects from preclinical and extrapolated strategies. 

**Table 3:** Evidence hierarchy for candidate LDN-related therapeutic strategies in rheumatic disease.

Strategy/example	Current evidence in rheumatic disease	Evidence tier	Interpretive limitation
JAK/IFN-pathway modulation (e.g., tofacitinib)	Human SLE study associated treatment with improved vascular/neutrophil-related abnormalities (86)	Human clinical, indirect	Drug is not LDN-specific; reduced LDN/NET phenotype may be secondary to inflammation control
IL-6 blockade (tocilizumab)	RA treatment associated with reduced LDN proportions/NETosis and improved endothelial function (95)	Human clinical, indirect	Does not establish direct pharmacological targeting of LDNs
Platelet TLR7/NET/mtROS pathways	Disease-proximal mechanistic evidence in SLE and lupus nephritis (29, 39, 87)	Patient-derived/preclinical mechanistic	Pathway involvement does not equal demonstrated therapeutic efficacy
CXCR2/chemotaxis, protease or PAD4-directed approaches	Predominantly general neutrophil or preclinical inflammatory-model literature (113–116)	Preclinical/extrapolated	Selectivity for pathogenic LDN states and clinical benefit remain unproven
Subset-selective depletion or metabolic/epigenetic reprogramming	No established LDN-specific therapy in rheumatic disease	Future concept	Requires validated disease-specific markers, causal evidence and safety data

### 4.2 Candidate therapeutic strategies and level of evidence

Candidate strategies can be grouped into modulation of LDN generation or trafficking, inhibition of pathogenic effector pathways, and future subset-selective depletion or phenotypic reprogramming (**Figure 8**). Their evidentiary support differs substantially, and none currently constitutes an approved LDN-specific therapy for rheumatic disease.

**Figure 8:**
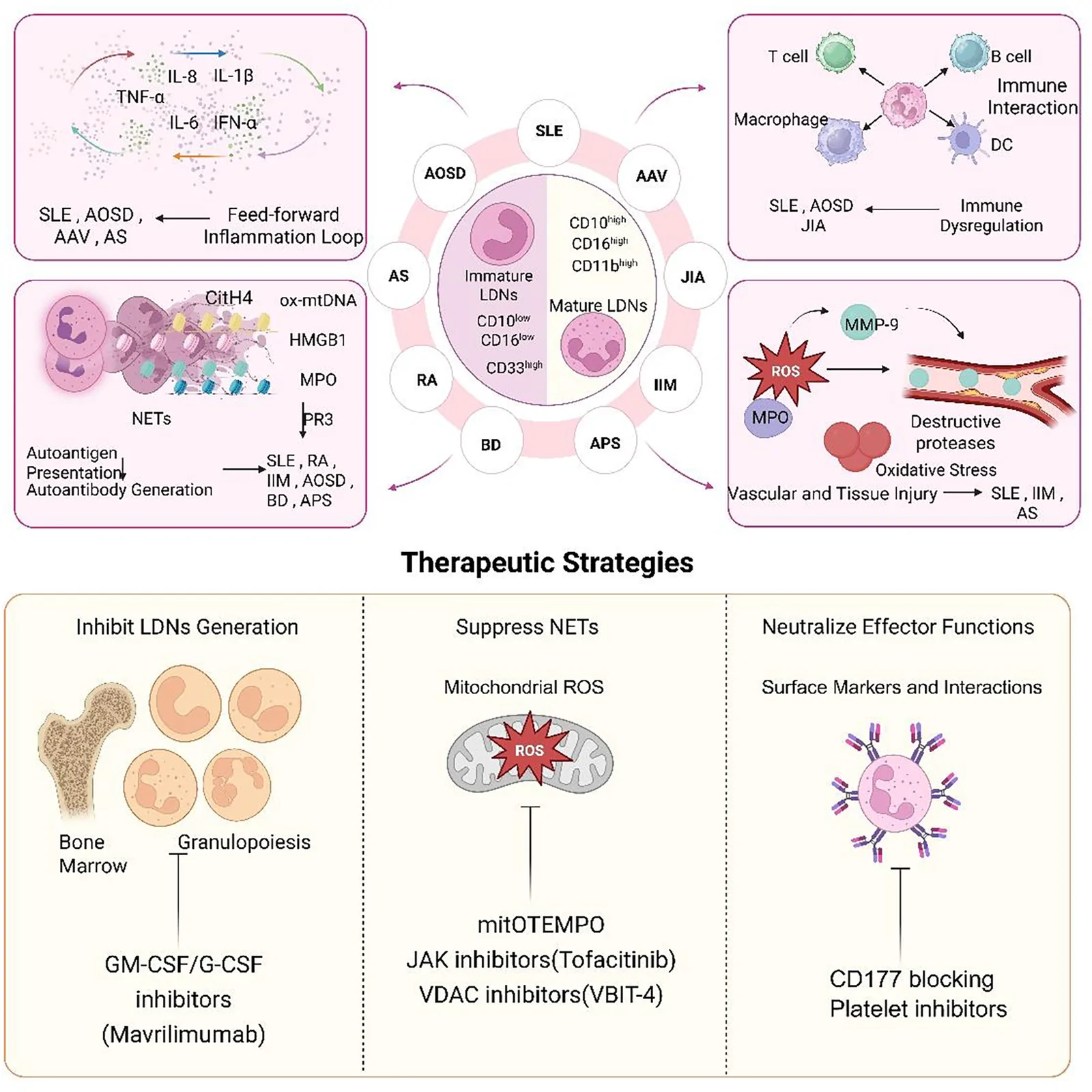
Disease-wide effects of heterogeneous LDN populations and candidate intervention points in rheumatic autoimmunity. The figure summarizes potential intervention points at the levels of granulopoiesis/trafficking, NET-associated effector pathways and cell-state modulation. The examples shown span different levels of evidence: some are supported by studies in rheumatic disease, whereas others are extrapolated from general neutrophil or non-rheumatic models and should be regarded as investigational concepts rather than established LDN-directed treatments. **Figure 8** Schematic diagram created with https://www.BioRender.com.

#### 4.2.1 Modulating LDN generation and trafficking

Inflammatory cytokines and chemokine pathways can influence granulopoiesis, neutrophil release and tissue recruitment. The CXCL8/CXCR2 axis is well established in general neutrophil trafficking biology (114, 115), and inhibition of chemokine signaling has been explored in multiple inflammatory settings. However, direct evidence that CXCR2, CCR2 or related interventions selectively suppress pathogenic LDN generation or tissue recruitment in human rheumatic disease is limited. These approaches should therefore be considered extrapolated or preclinical rather than validated LDN-specific strategies.

The principal translational concern is specificity. Pathways governing LDN accumulation also participate in normal antimicrobial defense and tissue repair, and currently available inhibitors do not distinguish low-density cells from conventional activated neutrophils (16, 115). Demonstration of reduced LDN numbers after pathway blockade would also require mechanistic confirmation that the effect is direct rather than secondary to reduced systemic inflammation.

#### 4.2.2 Inhibiting pathogenic effector pathways

Among proposed interventions, targeting disease-relevant effector pathways has the clearest mechanistic rationale. NET formation, mitochondrial ROS and immunostimulatory nucleic acids are implicated in SLE and other rheumatic diseases (29, 39, 87). Interventions directed at NET generation, NET clearance or upstream inflammatory signaling have shown proof-of-concept activity in cellular or animal studies, but most are not LDN-specific and few have been tested prospectively as therapeutic strategies in patients with rheumatic disease.

Claims for PAD4 inhibitors, CDK inhibitors, antioxidant approaches and neutrophil serine-protease inhibitors should therefore be calibrated to their experimental context. Some evidence derives from lupus or other autoimmune models, whereas much of the pharmacological literature on neutrophil proteases, chemotaxis and NET targeting comes from general neutrophil biology, airway inflammation or cancer (113, 116). These studies provide mechanistic plausibility but cannot be used as direct evidence of efficacy or safety for LDN-targeted therapy in rheumatic disease.

#### 4.2.3 Subset depletion and phenotypic reprogramming as future concepts

Selective depletion of a pathogenic LDN subset would require a marker that is both sufficiently specific and causally linked to a harmful cell state. At present, no surface marker uniquely identifies all pathogenic LDNs across rheumatic diseases, and markers such as CD177 primarily define enriched phenotypes rather than validated therapeutic targets. Antibody-based depletion, nanocarrier delivery and related cell-directed approaches are therefore largely conceptual or extrapolated from cancer and general neutrophil-targeting research (117–119).

Phenotypic or metabolic reprogramming is similarly promising but unproven. Epigenetic and metabolic studies demonstrate that disease-associated LDNs can have distinct transcriptional, epigenetic and metabolic states (36, 76), but these observations do not establish that such states can be safely and durably reversed in patients. Strategies targeting metabolism or epigenetic regulation should therefore be framed as future hypotheses requiring subset definition, mechanistic validation and disease-specific testing rather than as established therapeutic modalities.

### 4.3 Translational limitations

The first limitation is target definition. Because LDN is a density-based category containing immature, activated mature and potentially immunoregulatory populations, a treatment directed at the bulk low-density compartment risks combining biologically distinct targets (14). The absence of standardized isolation methods and subset-specific markers also makes reproducibility across laboratories and trials difficult.

The second limitation is the evidence gap. Most candidate interventions remain at the level of cellular experiments, animal models or extrapolation from other disease settings, and there is no established LDN-specific efficacy endpoint, dosing strategy or long-term safety framework. Species differences in neutrophil development and function further limit direct translation from mouse models to human chronic autoimmune disease.

The third limitation is biological duality. Broad neutrophil inhibition may impair host defense, and low-density populations may include states with neutral or regulatory functions. In addition, reductions in LDN abundance after successful treatment may reflect suppression of upstream inflammation rather than direct drug action. These considerations argue for pathway- and subset-resolved pharmacodynamic studies before LDN-directed claims are advanced clinically.

### 4.4 Future directions

Therapeutic development should proceed in parallel with improved biological classification. Priorities include reproducible molecular definition of pathogenic subsets, validation of necessity and sufficiency in disease-relevant models, paired blood-tissue pharmacodynamic studies, and prospective trials that incorporate LDN-related biomarkers without assuming that those biomarkers are themselves causal targets.

Until such evidence is available, it is most appropriate to describe LDN-directed approaches as candidate strategies. The strongest near-term opportunity may be to test well-defined disease-relevant effector pathways while preserving normal neutrophil function, whereas subset-specific depletion or reprogramming should remain a longer-term objective contingent on validated markers and safety data.

### 4.5 Summary

LDN biology provides several plausible therapeutic entry points, but the field has not yet established an LDN-specific treatment for rheumatic disease. Current evidence is strongest for disease-associated pathways and indirect modulation by established anti-inflammatory therapies; cell-selective depletion, trafficking blockade and phenotypic reprogramming remain predominantly preclinical or extrapolated concepts. Future translation will depend on molecular subset definition, causal validation and explicit separation of rheumatic-disease evidence from findings in other neutrophil contexts.

## 5 Outstanding questions and future directions

The first priority is to move beyond density alone as the principal biological definition through a staged standardization program. Near-term milestones are harmonized pre-analytics and isolation, consensus minimum phenotyping, functional requirements for PMN-MDSC designation, and inter-laboratory ring testing. The next stage is conversion of single-cell and multi-omic signatures into reproducible density-independent panels, followed by paired blood–tissue validation and prospective clinical qualification. Only after these analytical and biological milestones are met should universal reference ranges, companion diagnostics or trial endpoints be proposed.

Second, the developmental origin of low-density populations remains unresolved. Emergency granulopoiesis and peripheral reprogramming are not mutually exclusive, and their relative contributions are likely context dependent. Time-resolved single-cell studies, lineage tracing, paired bone-marrow/blood analyses and spatial profiling of target tissues are needed to link developmental trajectory to function rather than infer lineage from density or morphology alone (8).

Third, correlation must be separated from causation using an explicit driver–amplifier–bystander framework. In SLE, RA, JIA, psoriasis and most other polygenic diseases, associations between LDN abundance and disease activity cannot determine whether a defined LDN state initiates pathology, emerges in response to inflammation and then amplifies it, or simply reports emergency granulopoiesis and systemic inflammatory burden. Monogenic autoinflammatory disorders provide more genetically proximal evidence for involvement of neutrophil dysregulation, but even these models do not prove that low density is necessary or sufficient. Progress will require longitudinal pre-disease sampling where feasible, subset-specific perturbation, paired blood–tissue analyses, lineage-resolved models and interventions that test whether selective modification of a defined LDN state changes disease independently of global inflammation.

Fourth, functional heterogeneity must be incorporated into study design. Low-density neutrophils occur in healthy individuals (8), and suppressive or regulatory neutrophil states are documented in other biological contexts. Longitudinal sampling across active disease and remission, together with paired blood-tissue studies, will be necessary to determine whether apparently contradictory functions reflect distinct subsets, dynamic state transitions or tissue-specific adaptation.

Finally, clinical translation should be evidence tiered. Biomarker studies require standardized assays, reference ranges and prospective validation, while therapeutic studies should explicitly distinguish direct observations in rheumatic disease from preclinical evidence and extrapolation from tumor/MDSC or general neutrophil biology. The immediate goal is therefore not pan-LDN depletion, but identification of reproducible pathogenic states or effector pathways that can be targeted without compromising host defense.

### 5.1 Concluding remarks

LDNs have become an informative window into neutrophil heterogeneity in rheumatic and autoimmune disease, but their biological and clinical significance cannot be inferred from low density alone. Progress will depend on replacing density-based generalizations with molecularly defined subsets, testing causal roles experimentally, and calibrating biomarker and therapeutic claims to the level of evidence. This framework distinguishes the present Review from recent descriptive overviews and provides a focused path from association toward clinically actionable neutrophil biology.
